# A microbial natural product fractionation library screen with HRMS/MS dereplication identifies new lipopeptaibiotics against Candida auris

**DOI:** 10.21203/rs.3.rs-5802877/v1

**Published:** 2025-01-17

**Authors:** Gerard Wright, Xuefei Chen, Kalinka Koteva, Sommer Chou, Allison Guitor, Daniel Pallant, Yunjin Lee, David Sychantha, Shawn French, Dirk Hackenberger, Nicole Robbins, Michael Cook, Eric Brown, Lesley MacNeil, Leah Cowen

**Affiliations:** McMaster University; McMaster University; McMaster University; McMaster University; McMaster University; McMaster University; University of Toronto; McMaster University; McMaster University; McMaster University; University of Toronto; M.G. DeGroote Institute for Infectious Disease Research; McMaster University; McMaster University; University of Toronto

## Abstract

The rise of drug-resistant fungal pathogens, including *Candida auris*, highlights the urgent need for novel antifungal therapies. We developed a cost-effective platform combining microbial extract prefractionation with rapid MS/MS-bioinformatics-based dereplication to efficiently prioritize new antifungal scaffolds. Screening *C. auris* and *C. albicans* revealed novel lipopeptaibiotics, coniotins, from *Coniochaeta hoffmannii* WAC11161, which were undetectable in crude extracts. Coniotins exhibited potent activity against critical fungal pathogens on the WHO Fungal Priority Pathogens List, including *C. albicans*, *C. neoformans*, multidrug-resistant *C. auris*, and *Aspergillus fumigatus*, with high selectivity and low resistance potential. Coniotin A targets β-glucan, compromising fungal cell wall integrity, remodelling, and sensitizing *C. auris* to caspofungin. Identification of a PKS-NRPS biosynthetic gene cluster further enables the discovery of related clusters encoding potential novel lipopeptaibiotics. This study demonstrates the power of natural product prefractionation in uncovering bioactive scaffolds and introduces coniotins as promising candidates for combating multidrug-resistant fungal pathogens.

## Introduction

Fungal diseases represent a significant threat to public health, affecting over a billion people globally and resulting in more than 2.5 million deaths annually^1, 2^ surpassing mortality rates from tuberculosis and malaria.^3, 4^ Developing antifungal therapies is particularly challenging due to the overlapping cell components, as well as conserved metabolic and biochemical pathways between fungi and their human hosts, leading to a limited repertoire of available treatments for invasive fungal infections.^5^ The emergence of drug-resistant fungal pathogens, such as *Candida auris*, which has caused recent outbreaks in healthcare settings, further exacerbates this issue.^6, 7^ *C. auris* is recognized as a critical priority pathogen by the World Health Organization (WHO)^8^ and has been classified as an urgent threat by the US Centers for Disease Control and Prevention (CDC).^9^ *C. auris* isolates resistant to all existing drugs are increasingly common.^10^ Unlike other *Candida* species, *C. auris* efficiently colonizes the skin, leading to rapid nosocomial transmission and systemic infections with mortality rates of 40–60%.^7, 11^ The urgent need for novel antifungal drugs is critical to prevent further failures in controlling fungal infections within hospitals and healthcare facilities.

Natural products and their derivatives have been an invaluable source of therapeutic agents ranging from antibiotics to anticancer agents thanks to their structural novelty, chemical complexity, and intrinsic bioactivity; consequently, natural products hold promise as leads for new antifungal drug discovery.^12, 13^ The traditional ‘compound first’ discovery strategy using phenotypic cell growth inhibition screens of crude extracts of bacteria and fungi contributed to over half of the antibiotics and antifungal drugs in everyday use today.^14, 15^ However, the rediscovery of well-known chemical scaffolds, including antifungal classes such as the polyenes, is an increasing challenge given the phenotypic dominance of highly expressed common scaffolds in natural product extracts.^16, 17^

Owing to the rapid advancement of DNA sequencing technology,^18, 19^ genome sequences of natural product producers have revealed large numbers of untapped biosynthetic gene clusters (BGCs) of metabolites, predicting that traditional extract screens vastly undersample the available chemical space of natural products.^20^ A ‘genes first’ genome mining strategy, coupled with advanced molecular technologies, is leading to the discovery of novel chemical entities.^21, 22, 23^ However, predicting the biological activities of the natural products discovered based on bioinformatic analyses is difficult, even with known compound classes, which limits its application in drug development.

An orthogonal approach to exploring untapped natural product chemical space is by fractionating crude natural product extracts before biological testing, thus uncovering bioactive compounds that may be produced in small quantities or masked by other activities from the complex crude extracts.^24^ This approach typically improves the hit rate in phenotypic screens and shows enhanced biological activity due to improved screening performance (e.g., less viscous samples for robotic platforms), increased concentration of active components present as minor metabolites in crude extracts, and separation of redundant and ubiquitous nuisance compounds from less abundant metabolites.^25 26^ However, the reported fractionation approaches that use semipreparative HPLC methods are challenging to scale in academic settings due to the significant resources needed for the dedicated equipment and personnel to prepare the libraries.^27^

We developed a cost-effective prefractionation library (PFL) platform using medium-pressure reverse-phase separation that is better suited for deployment in an academic lab.^28^ Here, we report a pilot application of this platform for antifungal discovery targeting *Candida auris* and *Candida albicans*, coupled with tandem mass spectrometry (MS²) and genomic mining of the relevant biosynthetic gene clusters for rapid dereplication. Our results highlight the effectiveness of this strategy, enabling rapid identification and triage of known antifungal agents (e.g., enniatins, surfactins, tunicamycins) and prioritizing the discovery of a novel lipopeptaibiotic antifungal, coniotin, which exhibits broad activity against multidrug-resistant fungal pathogens and is phenotypically undetectable in the crude extract. Unlike channel-forming lipopeptides, coniotin targets the fungal cell wall by binding β-glucan, disrupting cell wall remodelling, and sensitizing resistant *C. auris* to caspofungin, with promising selectivity and a low potential for resistance development. The identification of the linear NRPS-PKS hybrid gene cluster and the proposed biosynthetic pathway of coniotin enable the discovery and optimization of related clusters and novel lipopeptaibiotics. Notably, the unusual NRPS adenylation domain specific for the charged amino acid Asp and the PKS responsible for the N-acyl chain are both critical for bioactivity. This work highlights the utility of the PFL platform and rapid MS² dereplication in identifying novel antifungal agents, positioning coniotin as a new chemical scaffold for targeting fungal cell wall integrity and advancing antifungal drug discovery against multidrug-resistant pathogens.

## Results

### Antifungal screening of a fractionated natural product library

**Figure 1:**
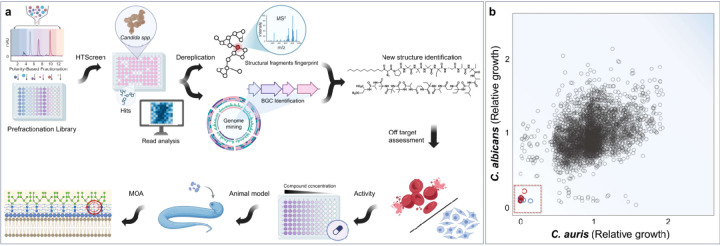
A scheme for the discovery of derisked novel antifungal natural products **a**, Overview of a rapid and risk-minimized approach for discovering novel antifungal natural products. The flowchart outlines a streamlined pipeline, starting from the construction of a prefractionation library (PFL), followed by high-throughput screening against target organisms (*Candida albicans* and *Candida auris*), rapid dereplication using tandem mass spectrometry (MS^2^) fingerprinting and bioinformatics analysis, structure determination, off-target assessment in mammalian cells (**HEK**: Human Embryonic Kidney cells + **RBC**: Red Blood Cell), broad-spectrum bioactivity evaluation, rapid therapeutic assessment using high-throughput animal models, and characterization of the mechanism of action (MOA). **b**, Scatter plots illustrating high-throughput screening results of crude methanolic extracts and PFL against *C. albicans* (y-axis) and *C. auris* (x-axis). Colored circles within the red box represent active hits: crude extracts (blue) or fractions (red) that inhibit Candida growth by at least 75% compared to the untreated control. Active hits with their WAC and fraction identities are shown in Extended Data Fig. 1a, with validation results in Extended Data Fig. 1b.

The PFL was derived from the medium-pressure reverse phase separation of fermentation methanolic extracts of our in-house collection of bacteria and fungi, resulting in eight fractions of metabolites sorted by hydrophilicity for each strain.^28^ A total of 3048 fractions and the corresponding 381 crude extracts were screened against *C. auris* CBS10913 in duplicate, identifying 43 hits that showed growth inhibition from fractions, while only 12 hits were from crude extracts (Fig. 1a). Similarly, a parallel screen against *C. albicans* ATCC90028 yielded 28 fractions and 9 crude hits. To identify broad-spectrum antifungal agents, nine hits shared across the two PFL screens were selected for further validation (Fig. 1b). Among these, antifungal bioactivity from WAC11084, WAC11113, and WAC11161 was exclusively observed in the fractionated samples, whereas their corresponding crude extracts showed minimal activity and were not identified during the cross-species hit screening (Fig. 1b, Extended Data Fig. 1a). These results highlight the advantage of screening fractionated extracts to improve hit detection.

#### Verification and rapid identification of known scaffolds.

The WAC isolates that produced the nine anti-*Candida* hits were successfully re-grown, extracted and re-fractionated to confirm their activity. The crude extracts were separated on a C18 Combiflash column, and 24 fractions were collected from each run, resulting in 216 fractions generated in approximately 4 h of instrument time (Extended Data Fig. 1b). All fractions were tested against *C. auris* CBS10913, but anti-*Candida* activity could not be reproduced for three of the nine isolates (WAC10997, WAC11024, and WAC11113), a common occurrence when re-evaluating wild actinomycete isolates from high throughput screening (Extended Data Fig. 1b).

**Figure 2:**
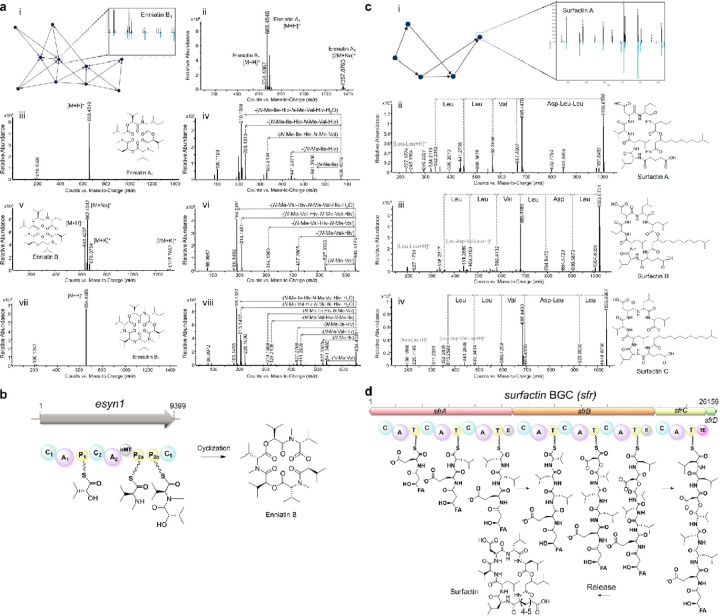
Rapid identification of known chemical scaffolds using high-resolution (HR) mass spectrometry coupled with bioinformatics analysis. **a,** Identification of enniatin from active fractions of WAC11175 through Global Natural Products Social Molecular Networking (GNPS) based on HRMS and MS/MS data. (i) The enniatin molecular network, with tandem MS fragment ion matches between the sample entity (blue) and reference enniatin B1 (black), is shown in the inset panel. (ii) HR-LCMS analysis for enniatins identified in active fractions of WAC11175, including the structures and representative HRMS spectra of enniatin A1 (iii), enniatin B (v), and enniatin B1 (vii). Accurate MS/MS spectra for enniatins A1, B, and B1, acquired at a single collision energy of 20 eV, along with fragmentation analyses, are shown in (iv), (vi), and (viii). N-Me-Val, N-Me-Ile, and Hiv represent N-Methyl-L-valine, N-Methyl-L-isoleucine, and 2-hydroxyisovaleric acid, respectively. b, Identification of the enniatin biosynthetic gene cluster (BGC) in the genome of WAC11175 and depiction of its biosynthesis. Esyn1, the enniatin synthetase, contains the following domains: C (condensation), A (adenylation), P (phosphopantetheine attachment site), and nMT (N-methyltransferase). The precursors, L-valine and D-hydroxycarboxylic acids, are activated at the A domain, and the building blocks are transferred between modules via P-domains. The final condensation, cyclization, and release from the enzyme are catalyzed by the C-domains. c, Identification of surfactin from active fractions of WAC11084. (i) GNPS molecular networking, constructed from HR-MS/MS data, showing tandem MS fragment ion matches (inset panel) between the identified entity (blue) and reference surfactin C (black). MS/MS fragmentation patterns and structures of surfactins are shown below: (ii) Surfactin A ([M + H]^+^, *m/z* 1008.6565) fragments into main ions at 667.4, 568.3, and 455.3; (iii) Surfactin B ([M + H]^+^, *m/z* 1022.6731) into 909.5, 681.4, and 582.4; (iv) Surfactin C ([M + H]^+^, *m/z* 1036.6907) into 923.6, 695.4, and 596.4. Additional fragment ions [M + H]^+^ confirm specific amino acid residue sequences, including *m/z* 227.1750 [Leu+Leu+H]^+^, 229.1145 [Asp+Leu+H]^+^, and 441.2699 [Leu+Asp+Val+Leu+H]^+^. d, Genetic organization of the surfactin BGC in WAC11084 and proposed biosynthesis. The surfactin synthetase complex consists of three modular units: SrfA, SrfB, mono-modular SrfC, and SrfD, responsible for synthesizing the seven amino acids of surfactin. Key domains include C (condensation), A (adenylation), T (thiolation), E (epimerization), and TE (thioesterase). The TE domain facilitates the release and cyclization of surfactin.

To rapidly identify known compound scaffolds and prioritize novel structures, high-resolution (HR) mass spectrometry (MS) coupled with tandem mass spectrometry (MS^2^) was used to characterize the structure of active metabolites. The MS and MS^2^ fragmentation spectra served as molecular fingerprints, enabling the construction of molecular networks that link to known compound classes by calculating spectral similarity relationships within the Global Natural Products Social molecular networking (GNPS, https://gnps.ucsd.edu/) platform (Fig. 2a).^29, 30^ A spectral alignment of precursor and fragment ions identified a series of candidate enniatin analogs from the active fractions of isolate WAC11175, which was confirmed to be a strain of *Metarhizium granulomatis* (Fig. 2a). The identified analogs included enniatin A1 ([M + H]^+^ at *m/z* 668.4519), enniatin B ([M + H]^+^ at m/z 640.4207), and enniatin B1 ([M + H]^+^ at *m/z* 654.4369) (Fig. 2a), which are well-known nonribosomal peptide mycotoxins^31^ now found to exhibit significant activity against *C. auris* (Extended Data Fig. 1b). Further supporting structure identification, the biosynthetic gene cluster (BGC) encoding the nonribosomal cyclic peptide synthetase for enniatin was identified from the genome sequence of WAC11175 (Fig. 2b, Supplementary Table 1).^32, 33^ To validate this proof-of-concept approach for coupling HRMS^2^ with bioinformatics-based rapid identification of active compounds, we purified enniatin B and confirmed its structure through NMR analysis, thereby verifying the reliability of the method (Supplementary Table 2, Supplementary Data 1).

Using this strategy, we identified the cyclic lipopeptides surfactin A ([M + H]^+^ at *m/z* 1008.6565), surfactin B ([M + H]^+^ at *m/z* 1022.6731), and surfactin C ([M + H]^+^ at m/z 1036.6907) in the active fraction from isolate WAC11084, identified as *Bacillus velezensis*, (Fig. 2c–d, Supplementary Table 1), whose antifungal activity had been masked in crude extracts (Extended Data Fig. 1a).^34^ The nonribosomal peptide synthetase (NRPS) cluster was identified in the genome sequence of WAC11084 and directs the synthesis of the amphiphilic cyclic lipopeptide, which imparts strong surfactant properties and broad-spectrum biological activities.^35^

Similarly, we observed that *Streptomyces microflavus* strains WAC1325 and WAC1490 produce tunicamycins, displaying anti-*Candida* activity in the active fractions (Extended Data Fig. 1b, 2a). Tunicamycins are well-established antifungal agents that inhibit the unfolded protein response by blocking protein N-glycosylation, thereby inducing ER stress in fungi.^36, 37^ Genome sequencing of WAC1325 and WAC1490 confirmed the presence of a tunicamycin biosynthetic gene cluster (BGC), supporting this discovery (Extended Data Fig. 2b, Supplementary Table 1).^38, 39^ We also detected the guanidinopolyol cyclic macrolides niphimycin C ([M + H]^+^ at *m/z* 1142.7335) and D ([M + H]^+^ at *m/z* 1228.7386) from active fractions produced by *Streptomyces antimycoticus* WAC5858 and confirmed the presence of the expected BGC in the sequenced genome (Extended Data Fig. 2c–e, Supplementary Table 1).^40^

#### Isolation and characterization of novel antifungal lipopeptaibiotics from the Coniochaeta fungus WAC11161.

**Figure 3:**
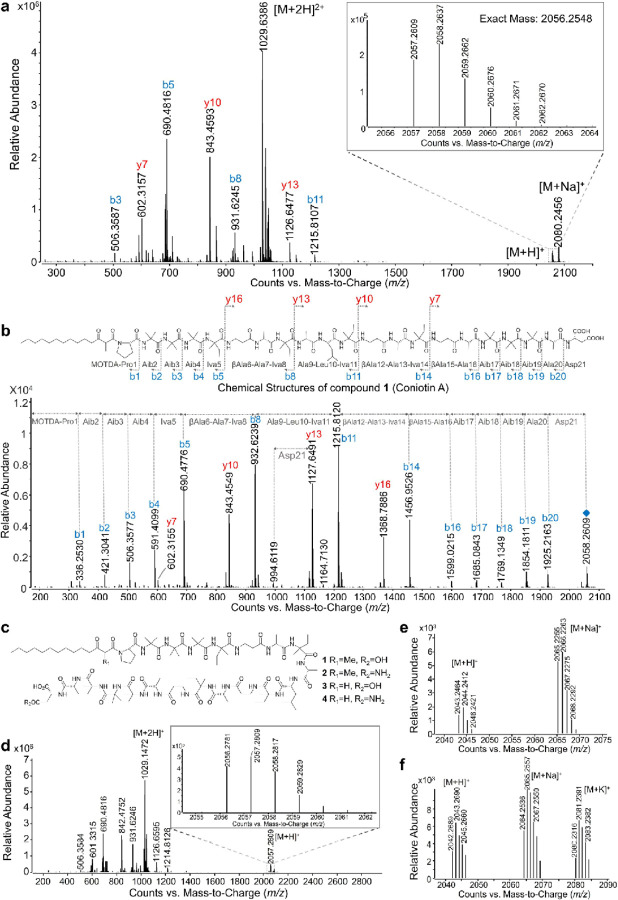
Characterization of novel antifungal lipopeptaibiotics from the *Coniochaeta* fungus WAC11161. **a**, High-resolution mass spectrum of compound **1** obtained with QToF mass spectrometry, showing the [M+H]^+^ ion at *m/z* 2057.2609. In-source fragmentation produced ions at *m/z* 506.36, 602.31, 690.48, 843.45, 931.62, 1126.65, and 1215.81. b, MS/MS analysis of compound 1 using collision-induced dissociation (CID) combined with a product ion scan (MS/MS) of nominal *m*/*z* 2057.26. Precursor ion indicated with a blue square. The structure of the lipopeptide 1 (termed coniotin A) is displayed above, along with its collision-induced fragmentation pattern, which corresponds to the detected b ions in the MS/MS spectrum. The b ions are labeled in blue, and the y ions are labeled in red. c, The structures of antifungal lipopeptaibiotics analogues 1, 2, 3, and 4, identified from the *Coniochaeta* fungus WAC11161, termed coniotin A, B, C, and D. d, High-resolution mass spectrum of coniotin B obtained with QToF mass spectrometry, showing the [M+H]^+^ ion at m/z 2056.2781 (calculated for C98H171N22O25, 2056.2780). e, High-resolution mass spectrum of coniotin C obtained with QToF mass spectrometry, showing the [M+H]^+^ ion at m/z 2043.2464 (calculated for C97H168N21O26^+^, 2043.2464) and the [M+Na]^+^ ion at m/z 2065.2255. f, High-resolution mass spectrum of coniotin D obtained using QToF mass spectrometry, showing the [M+H]^+^ ion at m/z 2042.2689 (calculated for C97H169N22O25^+^, 2042.2624), the [M+Na]^+^ ion at *m/z* 2064.2536 and the [M+K]^+^ ion at m/z 2080.2316.

In contrast to known compounds, the antifungal metabolites present in the active fractions of *Coniochaeta* fungal species WAC11161 could not be identified using HRMS data analysis in GNPS, suggesting the presence of unique structural features not previously characterized. Activity-guided isolation identified the active compound **1** ([M + H]^+^ at *m/z* 2057.2609) with a molecular formula of C_98_H_170_N_21_O_26_, as determined by HRMS (Fig. 3a). ^1^H NMR analysis revealed a peptide structure rich in highly methylated amino acids (Supplementary Table 3, Supplementary Data2), with further structural confirmation achieved through high-resolution mass fragmentation and Collision-Induced Dissociation (CID including MS^2^ and in-source CID, MS^3^).^41, 42^ CID-MS^2^ of the parent ion [M + H]^+^ at *m/z* 2057.26 and the doubly charged ion [M + 2H]^2+^ at *m/z* 1029.13 (Fig. 3a–3b, Extended Data Fig. 3i) identified characteristic fragment ions ([M-H_2_O + H]^+^ at *m/z* 85.05), indicative of rare non-proteinogenic α-aminoisobutyric acid (Aib), a hallmark of fungal peptaibiotics essential for their stable α-helical structures.^43 1^H-, ^13^C-, and 2-D NMR spectra (COSY, HSQC, HMBC, NOESY, ^1^H-^15^N-HSQC) identified six 2-Aib residues, five Ala, four Iva, three β-Ala, one Pro, one Leu, and one Asp residue, the latter being rare among peptaibiotics, with the 2-methyl-3-oxotetradecanoyl linked to the Pro residue (Fig. 3b, Supplementary Table 3, Supplementary Data 2).

In-source fragmentation produced ions at *m/z* 506.36, 602.31, 843.45, 931.62, 1126.65, and 1215.81 (Fig. 3a), with *m/z* sums of 931.62 + 1126.65 and 843.45 + 1215.81 matching the molecular weight of **1**, identifying them as N-terminal and C-terminal fragments, respectively. The fragment at *m/z* 336 was assigned to the prolyl-2-methyl-3-oxo-tetradecanoic acid moiety (Pro-MOTDA) (Fig. 3b), and the presence of the β-keto acid, 2-methyl-3-oxo-tetradecanoic acid (MOTDA), was further confirmed by analyzing an ethyl acetate extract of hydrolyzed **1**.^41, 44, 45^ CID-MS^2^ analysis of the parent ion (MS^2^), along with *N*-terminal, C-terminal and intermediate peptide fragment (MS^3^)^43^ identified the amino acid sequence as MOTDA-Pro-Aib-Aib-Aib-Iva-βAla-Ala-Iva-Ala-Iva-Leu-βAla-Ala-Iva-βAla-Ala-Aib-Aib-Aib-Ala-Asp-OH, a unique lipopeptaibiotic (Fig. 3b, Extended Data Fig. 3a–d). While α-cleavage of β-Ala ions was significantly suppressed,^46^ digestion with the non-specific protease papain generated peptide fragments ([M + H]^+^ at m/z 1303.85, 843.45, and 614.38), significantly facilitating sequence resolution (Extended Data Fig. 3b–d).^47^ Additionally, increasing CID to 50 eV enabled cleavage at βAla6-Ala7 and Ala9-Leu10, confirming the amino acid sequence at the single-residue level (Extended Data Fig. 3d–e). Amino acid stereochemistry was confirmed by Marfey’s analysis, with HPLC separation of the modified, hydrolyzed peptide revealing seven amino acids (Pro, Aib, βAla, Ala, Iva, Leu, and Asp) in **1** and identifying the absolute configurations as L-Pro, L-Ala, L-Leu, L-Asp, and D-Iva (Extended Data Fig. 3f).^48^

A second active analogue, compound **2** ([M + H]^+^ at *m/z* 2056.2781, [M + 2H]^2+^ at *m/z* 1028.64), was purified with a molecular formula of C_98_H_170_N_22_O_25_ (cal [M + H]^+^ at *m/z* 2056.2780) (Fig. 3c–3d, Extended Data Fig. 3i). The mass difference of 0.983 between compounds **1** and **2**, characteristic of -NH_2_ vs. -OH, suggests an Asn at the N-terminus instead of Asp. CID-MS^2^ analysis confirmed an identical amino acid sequence to **1**, with Asp replaced by Asn at the C-terminus (Extended Data Fig. 3g), which was further confirmed by Marfey’s analysis. Additionally, a non-methylated analogue, compound **3** ([M + H]^+^ at *m/z* 2043.2464, [M + 2H]^2+^ at *m/z* 1028.64) was identified (Fig. 3c, 3e, Extended Data Fig. 3j), featuring a 3-oxotetradecanoic acid (OTDA) at the N-terminus in place of **1**’s MOTDA (Fig. 3c). Each b fragment displayed an ion loss of 14 (-CH_3_ + H), including the terminal b1 fragment, identified as Pro-OTDA, at m/z 322 (Extended Data Fig. 3h). Similarly, analogue **4**, featuring an Asn at the N-terminus in place of Asp as in compound **3**, was also identified (Fig. 3c, 3f, Extended Data Fig. 3j). Accordingly, we designated this novel group of lipopeptaibiotics, **1**, **2**, **3**, and **4**, derived from the *Coniochaeta* fungi as coniotin A, B, C, and D.

#### Coniotins exhibit selective antifungal activity.

**Figure 4:**
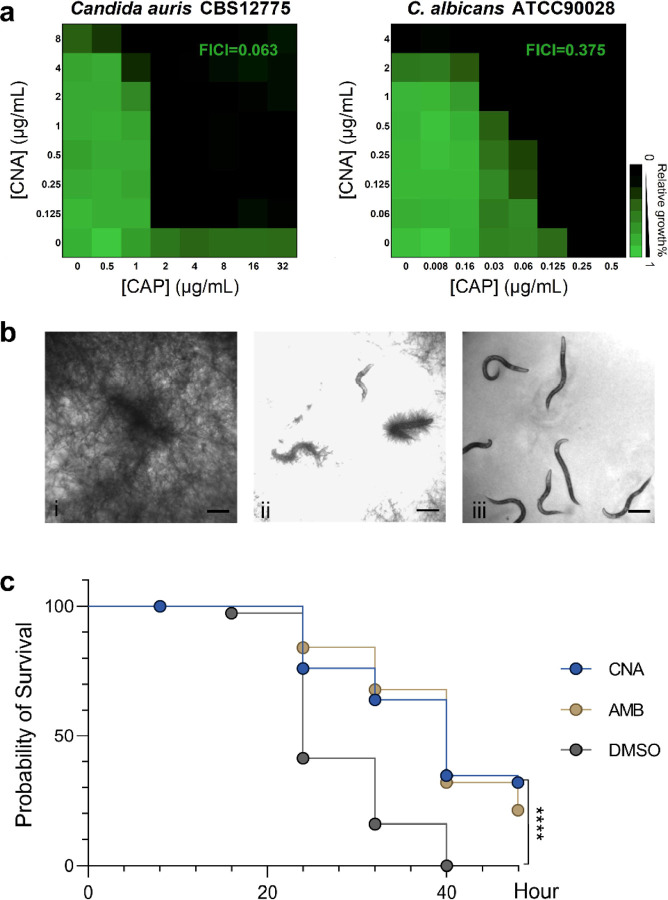
Antifungal activity of coniotin against *C. albicans* and multidrug-resistant *Candidaauris* **a**, Coniotin A (CAN) synergizes with caspofungin (CAP) in *Candida* species. Checkerboard assays depicted as heatmaps show the average growth of biological duplicates, normalized to controls without compounds. The potentiation of coniotin A and caspofungin was evaluated against *C. auris* CBS12775 and *C. albicans* ATCC90028. Relative growth is depicted by colour, as indicated by the scale bar in the bottom right. Fractional Inhibitory Concentration Index (FICI) values, calculated as described in the Methods, are shown in the top right corner of each checkerboard. FICI values below 0.5 denote synergistic interactions. **b**, Rapid assessment of the therapeutic potential of coniotin A using high-throughput phenotypic screening in a *Caenorhabditis elegans-Candida albicans* infection model. *C. elegans* were infected with *C. albicans* ATCC90028 and treated with various concentrations of coniotin A. Representative images show worms treated with DMSO (i, negative control), 1 µg/ml coniotin A (ii), and 8 µg/ml coniotin A (iii). Scale bar = 0.2 mm. **c**, Survival of *C. elegans* infected with *C. auris* CBS 12775 and treated with amphotericin B (AMB), coniotin A (CNA), or vehicle dimethyl sulfoxide (DMSO). Twenty-five worms per condition were observed over a 48-hour period in three independent trials. Survival was analyzed using Kaplan-Meier survival curves, and statistical significance was determined by the Log-rank (Mantel-Cox) test, comparing CNA (1× MIC) treatment to the DMSO control group, with p-values reported as **** for p < 0.0001.

Lipopeptides exhibit a wide range of biological activities due to their unique structures.^49, 50^ We assessed the antifungal activity of coniotin A in comparison to first-line antifungals (caspofungin, amphotericin B, and fluconazole), revealing broad-spectrum activity against *Candida* species (*C. albicans*, *C. parapsilosis*, and *C. tropicalis*), *Cryptococcus neoformans*, *Nakaseomyces glabratus* and *Saccharomyces cerevisiae* (Table 1). Notably, coniotin A demonstrated potent activity against multidrug-resistant *C. auris* and the mold *Aspergillus fumigatus*, both identified as critical threats on the WHO Fungal Priority Pathogens List^8^, surpassing the efficacy of caspofungin and fluconazole. Interestingly, coniotin A also enhanced the efficacy of caspofungin, significantly reducing its MIC against refractory *C. auris* to the CLSI breakpoint of 2 µg/mL (Fig. 4a).^51^

The therapeutic potential of coniotin A was rapidly assessed using the non-mammalian model *Caenorhabditis elegans*, a system well-suited for studying *Candida* interactions due to its intestinal similarities to mammals and ease of infection.^52^ At a concentration of 8 µg/mL, coniotin A significantly reduced *C. albicans* infections in *C. elegans* (Fig. 4b). Building upon these results, further *in vivo* efficacy was evaluated against multidrug-resistant *C. auris*.^53^ Coniotin A effectively extended the lifespan of *C. elegans* pre-infected with *C. auris* CBS 12775, a strain resistant to caspofungin and fluconazole, by approximately 30% over two days, while untreated nematodes succumbed to infection within 40 hours (Fig. 4c). Transmission electron microscopy (TEM) demonstrated the interactions between the pathogen and host during infection, revealing that *C. auris* invaded through the intestinal wall, resulting in the disintegration of the brush border of the gastrointestinal tract (Extended Data Fig. 4a).^54^ Unlike membrane-perturbing lipopeptides such as iturin A,^55^ coniotin A exhibited no hemolytic or antibacterial activity, indicating a different mode of action, with its target being absent in prokaryotes and human erythrocytes (Table 1, Supplementary Table 4, Extended Data Fig. 4b).

Interestingly, amidation of the C-terminal Asp slightly reduced the antifungal efficacy of coniotin B against *C. albicans* ATCC 90028, *C. neoformans* H99, and *C. auris* strains CBS12766 and CBS12776 (Table 1). Furthermore, coniotin B demonstrated greater hemolytic and cytotoxic activity compared to coniotin A (Table 1, Extended Data Fig. 4b–c), highlighting the critical role of Asp in influencing its bioactivity^56^, despite the rarity of Asp residues in peptaibiotics due to their unfavorable effects on α-helix stabilization.^57^

#### Coniotin A targets β-glucan impairing cell wall integrity.

Fungal lipopeptaibols^58^ uniquely contain the nonstandard amino acids Aib, which confer an α-helix structure^59^ that enhances bioactivity and metabolic stability, allowing them to form ion channels, permeabilize cell membranes, and act as active agents.^60, 61^ To elucidate the mechanism of action for coniotin A, a serial passage assay was performed to select *C. albicans* ATCC90028 and *C. neoformans* H99 mutants resistant to coniotin A, as resistance mutations typically arise in target genes.^62^ However, after 20 serial passages under sub-MIC conditions, no resistance emerged, and all colonies remained susceptible to coniotin A, suggesting a low mutation rate or unstable resistance. Similarly, screening over 5,000 *C. albicans* heterozygous deletion mutants revealed no strains resistant to coniotin A (Extended Data Fig. 5a), suggesting it, like amphotericin B, may target essential cellular components rather than specific protein targets, making resistant mutants exceedingly rare and its precise target(s) unclear.^63^

**Figure 5:**
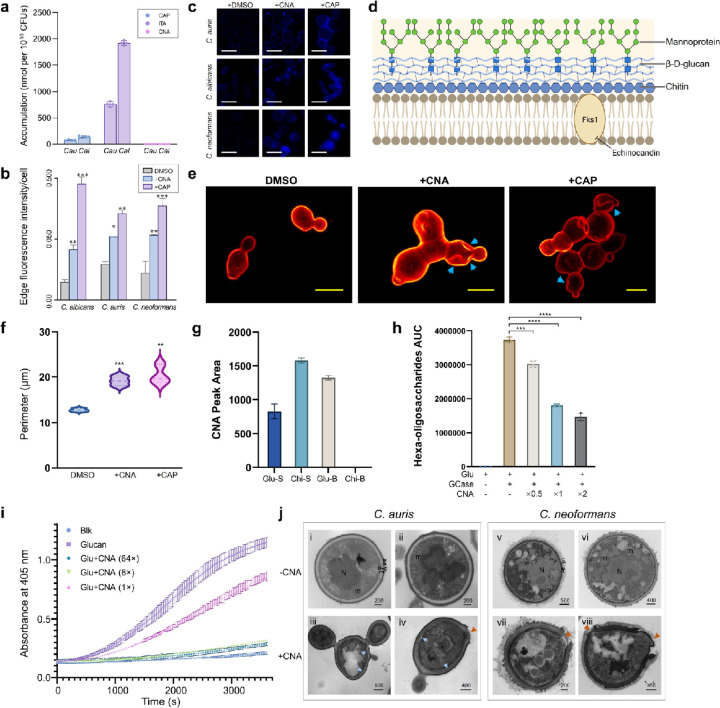
Coniotin A targets β-glucan impairing cell wall integrity. **a**, Intracellular accumulation of coniotin A (CNA), caspofungin (CAP) and iturin A (ITA) in *C. auris* CBS10913 (*Cau*) and *C. albicans* ATCC90028 (*Cal*) were quantified after 10 minutes of treatment. Surface-bound compound was removed using silicone oil prior to analysis. Results are expressed as the mean ± SD from three independent biological replicates. **b**, Increased cell wall chitin levels following coniotin A (CNA) and caspofungin (CAP) treatment. Quantification of calcofluor white (CFW) staining was performed to assess total cell wall chitin levels in log-phase cells of *C. albicans* ATCC90028, *C. auris* CBS10913, and *C. neoformans* H99. Cells were treated for four hours with half the MIC of CNA, CAP, or vehicle control (DMSO), stained with CFW, and imaged using a Nikon Eclipse Ti inverted microscope. Fluorescence intensity per cell was quantified using ImageJ and CellProfiler, analyzing 100 cells from at least three images per condition. Data are presented as the log mean edge fluorescence intensity ± SEM. Statistical significance was assessed using one-way ANOVA followed by Dunnett’s multiple comparisons test, with each treatment group compared to its respective DMSO control (*P < 0.05; ** P < 0.01; ***P < 0.001). c, Representative images of CFW staining in treated fungal cells related to Fig. 6b. Representative images showing *C. albicans* ATCC90028, *C. auris* CBS10913, and *C. neoformans* H99 cells treated with half the MIC of coniotin A (CNA), caspofungin (CAP), or vehicle control (DMSO), followed by CFW staining to assess chitin content. In CNA- and CAP-treated cells, bright, thickened septa were observed, forming proximal to the normal location at the mother-bud neck region. Scale bar = 10 µm. d, Structural organization and composition of *Candida* yeast cell wall. The outer cell wall of *Candida* yeasts is enriched with highly mannosylated proteins, predominantly anchored to the β-glucan and chitin core via glycosylphosphatidylinositol (GPI) remnants. Echinocandins target glucan synthase Fks1, a key enzyme displayed in the cell membrane, which is essential for the synthesis and integrity of the cell wall. e, The mannoprotein component of the fungal cell wall was fluorescently labelled with ConA-Alex647. *C. albicans* ATCC90028 cultures were grown to the mid-log phase in the presence of coniotin A (CNA), caspofungin (CAP), or vehicle control (DMSO) and stained with ConA-Alex647 for 10 minutes. Z-stack images were acquired using a Zeiss LSM980 Inverted Confocal Microscope using a 63×/1.4 oil-immersion objective. 3D projections of the yeast cells were generated in ImageJ. Blue arrows indicate cell wall damage. Scale bars = 5 μm. f, Perimeter of mid-log phase *C. albicans* ATCC90028 cells grown in SDB medium at 30°C in the presence of coniotin A (CNA), caspofungin (CAP), or vehicle control (DMSO). The cell periphery was visualized by staining with ConA-Alex647, and the perimeter and diameter were measured and analyzed using ImageJ by examining ~150 cells. Statistical significance was evaluated using two-tailed pairwise Student’s t-tests (**P < 0.01; ***P < 0.001). g, Quantitative analysis of coniotin A (CNA) binding in the pull-down assay. β−1,3-glucan (1 mg/mL) or chitin (1 mg/mL) was incubated with 32 µg/mL coniotin A (CNA) in PBS for 1 hour. Following incubation, the polysaccharides were collected, washed, and extracted with DMSO for analysis. CNA bound to β−1,3-glucan (Glu-B) or chitin (Chi-B), or remaining in the supernatant of β−1,3-glucan (Glu-S) or chitin (Chi-S) solutions, was quantified using high-resolution mass spectrometry. The Y-axis shows the relative abundance of CNA based on MS peak area, and the X-axis indicates the sample groups. Data are presented as mean ± SD from three independent biological replicates. h, Inhibition of β−1,3-glucan (Glu) digestion by coniotin A (CNA). Relative abundance of glucanase (GCase) digestion products (β−1,3-linked oligosaccharides: hexa-glucose) from 125 µg/mL laminarin (a β−1,3-glucan), incubated with glucanase for 0.5 h in the absence or presence of different concentrations of coniotin A (64 µg/mL, ×0.5; 128 µg/mL, ×1; 256 µg/mL, ×2). Presence is indicated as “+” and absence as Data were acquired using high-resolution mass spectrometry and presented as mean ± SD from triplicate runs. Statistical significance was determined using an unpaired t-test with Welch’s correction, comparing each coniotin A treatment to untreated controls. p-values: *** < 0.001, **** < 0.0001. i Kinetic curves of β-glucan activation of limulus coagulation factor G. The kinetic chromogenic reaction specific to (1,3)-β-D-glucan (Glu) using Glucatell^®^ kits: 100 pg/mL β−1,3-glucan was preincubated with or without coniotin A (CNA) at concentrations of 0.625 µg/mL (1×), 5 µg/mL (8×), and 40 µg/mL (64×). The samples were mixed with 100 µL reconstituted Glucatell reagent containing limulus coagulation factor G, and analyzed in a preheated plate reader at 37°C for 1 hour. The rate of change (mAbs/30s) was measured at 405 nm to determine intact (1,3)-β-D-glucan abundance. j, Transmission Electron Microscopy (TEM) images of *C. auris* CBS12766 and *C. neoformans* H99 showing abnormal cell wall structures following coniotin A (CNA) treatment. Cells were cultured with (+) and without (−) half MIC of CNA, fixed, and visualized via TEM. Vehicle (DMSO)-treated *C. auris* cells are shown in (i, ii), CNA-treated cells in (iii, iv), similarly, vehicle (DMSO)-treated *C. neoformans* cells in (v, vi), and CNA-treated *C. neoformans* (2 µg/mL) in (vii, viii). Observed cell wall defects in CNA-treated cells include detached membranes (blue arrowheads), compromised cell wall integrity (orange arrowheads), and abnormally increased thickness. Brackets denote distinct cell wall layers: G+C, β-glucan and chitin; M, mannoproteins. Additional cellular structures are labeled: nucleus (N) and mitochondria (m). Scale bars are depicted in each image, with units in nanometers (nm).

To investigate the target location of coniotin A and determine whether it requires intracellular entry to exert its activity, we first assessed its intracellular accumulation.^64^ The membrane- and cell wall-perturbing lipopeptide iturin A^55^ and β-(1,3)-glucan synthase inhibitor caspofungin^65^ were used as controls, both demonstrating measurable accumulation, with caspofungin displaying significantly lower levels (Fig. 5a). In contrast, no intracellular accumulation of coniotin A was detected in either pathogen, indicating that it exerts its activity at the cell surface rather than intracellularly (Fig. 5a). Chitin is an essential component of the fungal cell wall located in the inner layer and has tightly regulated synthesis that can be induced in response to β-glucan damage, aiding in survival against cell wall stressors.^66, 67, 68^ The levels of chitin were significantly elevated in response to coniotin A in *C. albicans*, *C. auris*, and *C. neoformans*, similar to the response observed with the β-(1,3)-glucan synthase inhibitor caspofungin (Fig. 5b–d). In addition to increased chitin production and thickened septa, treatment with coniotin A altered the morphology of *C. albicans*, leading to clusters of enlarged and elongated cells (Extended Data Fig. 5b).

Further visualization of the impaired cell surface was achieved by staining the outermost mannoprotein layer with Alexa594-ConA (Fig. 5d).^69^ The compromised cell wall in *C. albicans* treated with coniotin A and caspofungin was evident, as characterized by a collapsed cell surface and the simultaneous production of multiple daughter cells that failed to complete division (Fig. 5e), deviating from the normal unipolar budding where a single mother cell generates one daughter cell at a time.^70^ Cells treated with coniotin A exhibited a wide neck and morphological heterogeneity, aligning with characteristics and phenotypes commonly observed in cells exposed to cell wall-targeting agents, such as caspofungin (Fig. 5e).^71^ These observations indicate impaired cell wall remodeling, accompanied by disruptions in the structure and composition of the cell wall. Furthermore, treatment with coniotin A induced a 1.5-fold increase in the cell perimeter, based on analysis of over 150 stained cells (Fig. 5e–f); similar to caspofungin, this suggests cell wall softening, likely due to reduced β−1,3 glucan content, which compromises *C. albicans* cell shape, mechanical rigidity, and osmotic resistance, resulting in swollen cells.^72^

Given similar physiological effects with caspofungin, we evaluated the impact of coniotin A on β−1,3 glucan levels using aniline blue staining.^73, 74^ This analysis revealed a significant reduction in the staining of cell surface glucan, especially in characteristic cell wall regions (Extended Data Fig. 5c). This reduction was accompanied by increased diffusion of the stain into the cells, indicating enhanced osmotic fragility,^72^ suggesting that coniotin A primarily targets glucan fibrils, destabilizing polysaccharides and ultimately leading to cell wall damage. The direct interaction between coniotin A and β−1,3 glucan was confirmed through a pull-down assay. Over 50% of coniotin A bound to β−1,3 glucan within a 1-hour incubation, whereas chitin did not bind coniotin A, leaving a greater amount of it in the supernatant (Fig. 5g). To further validate this interaction, a BODIPY fluorescent moiety was conjugated to the terminal Asp of coniotin A, enabling visualization of its binding to β−1,3-glucan via a pull-down assay, with glucan particles analyzed through aniline blue staining (Extended Data Fig. 5d).

The binding of coniotin A to glucan further hindered the interaction of other enzymes or factors with glucan, as evidenced by its dose-dependent inhibition of glucanase-mediated glucan digestion, resulting in decreased production of hydrolyzed short-chain oligosaccharides (Fig. 5h). Similarly, coniotin A inhibited the activation of limulus coagulation factor G, which is highly sensitive to (1,3)-β-D-glucan (Fig. 5i). Typically, glucan-bound factor G initiates the coagulation cascade, generating the detectable chromophore p-nitroaniline (pNA) from the chromogenic substrate Boc-Leu-Gly-Arg-pNA. However, incubating with 5 µg/mL coniotin A significantly reduced reaction dynamics, suggesting a decrease in free, intact glucan. This effect likely results from the interaction of coniotin A with glucan, which may disrupt its single helical conformation, a key contributor to the activation of limulus coagulation factor G (Fig. 5i).^75^ Collectively, these findings suggest that the binding of coniotin A to glucan interferes with its enzymatic modification and remodeling during biophysical processes, ultimately leading to a functionally compromised cell wall.

Transmission electron microscopy (TEM) was used to further examine the morphological changes in cell wall structure under treatment with coniotin A. *Candida* cells typically display characteristic two-layered cell walls,^66, 76^ as seen in *C. auris* CBS 12766 control cells: an electron-dense, mannan-rich outer layer (M) and a glucan-rich inner layer of lower electron density (G + C) (Fig. 5j, i–ii), both continuous with the plasma membrane.^77, 78^ In cells treated with coniotin A, a predominance of a thicker, more electron-dense layer was observed in place of the translucent inner layer, with the cell wall detaching from the membrane (Fig. 5j, iii–iv), likely due to upregulated chitin production. Chitin appeared in the outer and inner wall layers, with cell wall proteins increasingly linked to chitin rather than β−1,3-glucan, as seen in caspofungin-treated, glucan-compromised cells.^66^ *C. neoformans* cells have distinct cell wall structures, with an exopolysaccharide capsule (C) anchored to the outer layer, which contains both α-glucan and β-glucan. In contrast, the inner layer is primarily composed of β-glucans and chitin. These two layers are tightly interwoven, forming a dense, thin cell wall (W) closely adjacent to the membrane (Fig. 5j, v–vi).^77^ Despite capsule protection, coniotin A induced severe cell wall damage, along with a much thicker, highly electron-dense cell wall structure, suggesting activation of chitin salvage pathways to withstand cell wall stress (Fig. 5j, vii–viii). Abnormal multilayered cell walls and focal enlargements were also observed in both *C. auris* and *C. neoformans* under treatment with coniotin A, suggesting that cell wall stress led to aberrant thickening as a survival response (Extended Data Fig. 5e, i–iv). Although *C. neoformans* possesses robust mechanical barriers that resist caspofungin, maintaining an intact cell wall and capsule even when killed by amphotericin B, cells killed by coniotin A exhibited an aberrantly thickened cell wall with clear signs of disintegration (Extended Data Fig. 5e, v–vii). TEM analysis of cell wall damage morphology reveals that coniotin A disrupts the dynamic physiological activity of the cell wall, leading to structural compromise, likely due to its targeting of glucan.

### Coniotins are generated by a hybrid PKS-NRPS biosynthetic gene cluster

**Figure 6:**
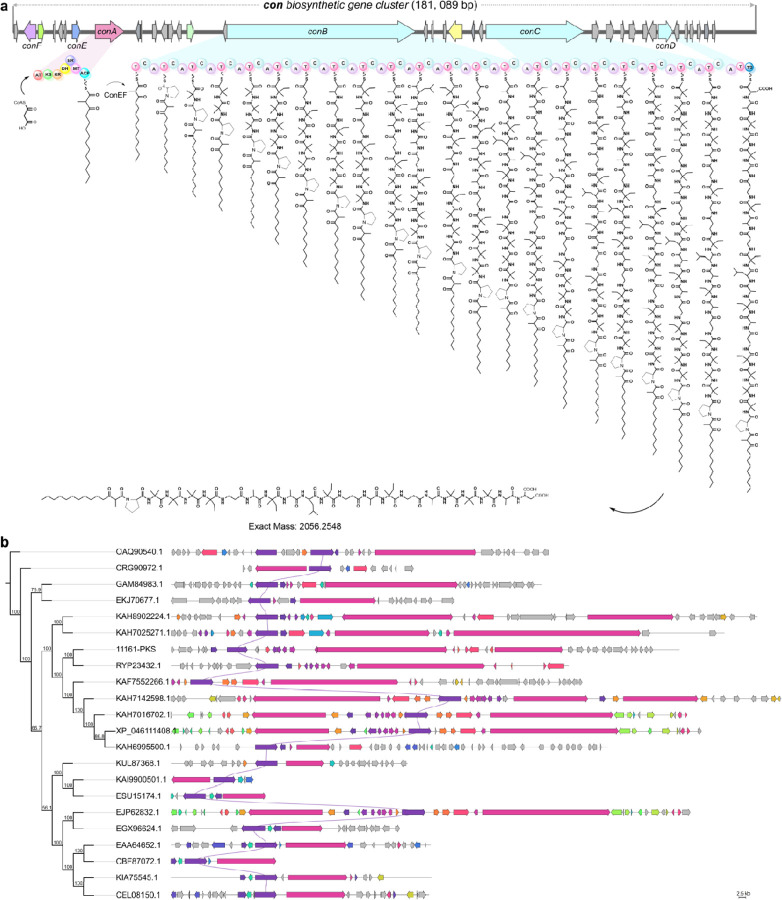
The biosynthetic gene cluster and proposed biosynthetic pathway of coniotin A. **a**, Organization of the coniotin biosynthetic gene cluster and proposed pathway in Coniochaeta hoffmannii WAC11161. Open reading frames (ORFs) involved in coniotin biosynthesis are color-coded as follows: pink for the polyketide synthase (PKS) gene (conA), blue for the non-ribosomal peptide synthetase (NRPS) genes (conB-D), dark blue for the acyl-CoA ligase gene (conE), light purple for the transferase gene (conF), light yellow for the ABC transporter, and green for potential functional genes. For ORF annotations, see also Supplementary Table 5. The PKS domains in ConA are labeled as follows: AT (acyltransferase), KS (keto synthase), ACP (acyl carrier protein), KR (ketoreductase), DH (dehydratase), ER (enoylreductase), and MT (methyltransferase). The NRPS domains in ConB-D are labeled as follows: C (condensation domain), A (adenylation domain), T (thiolation domain), and TD (terminal domain). **b**, A phylogenetic tree of ConA PKS analogs was constructed using the Jukes-Cantor genetic distance model and the neighbor-joining method, based on amino acid sequences of 22 ConA homologues (Left panel). To ensure statistical robustness, bootstrap resampling with 1,000 replicates and a random seed of 996327 was performed. Bootstrap values are displayed next to the nodes, and the ConA homologues are labeled with their respective protein IDs. The gene organization of biosynthetic gene clusters containing the corresponding ConA PKS analogs is shown on the right, compared and aligned with the phylogenetic tree. Protein-coding genes adjacent to the PKSs within these clusters are depicted as colored arrows, indicating transcriptional orientation. Genes with similar functions are color-coded for clarity: purple arrows denote ConA PKS analogs, with connecting linkers indicating sequence alignment identities exceeding 30%; pink arrows highlight NRPS genes.

Understanding the biosynthesis of coniotin is crucial for exploring, developing, and optimizing this novel class of fungal lipopeptaibiotics, as its bioactivity and structural stability are closely tied to key structural features, including N- and C-terminal modifications,^79^ the number and charge of amino acids,^80^ and peptide length.^81, 82^ Natural lipopeptides are typically synthesized by large multi-modular NRPSs, with the number of modules determining peptide length and the types of amino acids incorporated. However, fungal lipopeptide biosynthetic pathways remain relatively underexplored.^83^ Coniotins, containing 21 amino acids, are relatively rare among lipopeptaibiotics^79^ and produced by *Coniochaeta hoffmannii*, an ascomycete fungal plant pathogen with largely uncharacterized secondary metabolites. Therefore, we sequenced the genome of the producer strain WAC11161, identifying a hybrid NRPS-polyketide synthase (PKS) biosynthetic gene cluster (BGC, termed *con*) associated with coniotin, among 38 genomic BGCs predicted by antiSMASH^84^ (Fig. 6a, Supplementary Table 5). The *con* cluster comprises three NRPS genes (*conB*, *conC*, and *conD*), containing a total of 21 modules, each incorporating a specific amino acid to produce the 21-residue peptide. An upstream gene, *conA*, encodes an iterative type I PKS that repeatedly uses specific enzyme domains to assemble the N-terminal fatty acyl moieties of coniotin.

Based on the annotation of identified *con* BGC (Supplementary Table 5), the biosynthetic pathway of coniotin A is proposed as follows (Fig. 6a): The PKS (ConA) initiates synthesis by using its ketoacyl synthase (KS) domain to condense malonyl-CoA building blocks, delivered by the acyltransferase (AT) and tethered to the acyl carrier protein (ACP) domain, elongating the nascent polyketide chain by two-carbon units. As a highly reducing PKS, three β-keto processing domains: dehydratase (DH), enoyl reductase (ER), and ketoreductase (KR), drive selective reduction during each cycle, forming a highly saturated 3-oxotetradecanoyl structure that ultimately yields coniotin C and D. In contrast, β-C-methylation by the methyltransferase (MT) domain during the final cycle produces methylated polyketides, generating the major products coniotin A and B (Fig. 6a). The polyketide intermediates (OTDA/MOTDA), released from ConA, are converted to CoA thioesters by acyl-CoA ligase (ConE) and transferred to the initial thiolation (T_0_) domain of the NRPS (ConB).^85^ The first condensation domain (C_1_) catalyzes C-N bond formation, loading the initial prolyl thioester to produce the Pro-(M)OTDA moiety. Sequential incorporation of 20 additional amino acids extends the linear peptide chain, which is ultimately released by the terminal (TD) domain to complete the biosynthesis (Fig. 6a).

The N-terminal polyketide acyl moiety is critical for lipopeptide bioactivity,^86, 87^ with its length, saturation, and branching significantly influencing potency, complexity, and specificity. This structural diversity is shaped by polyketide synthases (PKSs), which iteratively employ a single set of catalytic domains, selectively engaging reduction and modification domains during each elongation cycle at specific positions.^88, 89^ This inherent flexibility allows PKSs to produce multiple lipopeptide isoforms from a single synthetase.^90^ Using the NCBI genome database (https://www.ncbi.nlm.nih.gov/genome/), we identified several PKS genes similar to *conA* and constructed a PKS consensus tree (Fig. 6b). These genes reside within hybrid PKS-NRPS BGCs (Fig. 6b)^91^, potentially involved in fungal lipopeptide biosynthesis, including known BGCs for beauveriolide and leucinostatin (Fig. 6b, EJP62832.1 and OAQ90540.1), as well as emericellamide-related clusters potentially encoding structurally similar lipopeptaibiotic analogues (Fig. 6b, GAM84983.1, EAA64652.1). The discovery of these and previously uncharacterized hybrids expands our understanding of fungal secondary metabolism and highlights the potential for uncovering novel lipopeptaibiotics with unique bioactivities.

## Discussion

Natural products and their derivatives have long served as an invaluable reservoir of therapeutic agents, contributing to nearly half of all approved anticancer drugs, due to the structural novelty, diversity, and complexity of their metabolites. Microorganisms play a pivotal role in antibiotic biosynthesis to gain a competitive advantage for survival. The availability of extensive microbial genome sequence databases has unveiled a vast reservoir of untapped biosynthetic gene clusters (BGCs) in microorganisms, revealing an immense, largely unexplored chemical space. However, the frequent rediscovery of known compounds poses a significant challenge to identifying novel bioactive molecules.

To overcome these challenges, we developed a cost-effective and resource-efficient platform to uncover active agents masked within crude extracts that are often overlooked using traditional screening methods, achieving over a 50% increase in hit rate. The prefractionated library effectively separates growth-enhancing and antibiotic molecules, as well as major and minor components, facilitating the detection of minimal or negligible secondary metabolite production. By leveraging an extensive and rapidly expanding database of annotated tandem mass spectrometry (MS/MS) fragmentation spectra and characterized biosynthetic gene clusters, we developed a rapid dereplication strategy integrated with PFL screening to prioritize novel chemical scaffolds. This approach enabled the identification of coniotins, a novel lipopeptaibiotic family distinguished by 21 amino acids and a 2-methyl-3-oxotetradecanoyl N-terminus. Notably, microbial lipopeptaibiotics with peptide chains exceeding 20 amino acids are rarely discovered. Their identification often requires advanced techniques such as genome mining and heterologous expression, as microorganisms tend to prioritize energy and resources for growth and maintenance under laboratory conditions, resulting in scarce biosynthesis of such complex secondary metabolites. Our strategy has demonstrated effectiveness in uncovering uncharacterized, microbially derived chemical structures, even from limited yields, thereby expediting the discovery and characterization of unique bioactive molecules for therapeutic applications.

The novel mechanism of action of coniotin A, which specifically targets β-glucan, enriches the limited antifungal arsenal while offering a resistance-aversive strategy through its ability to compromise the fungal cell wall. This disruption facilitates caspofungin’s access to its target, enabling synergistic antifungal activity. Long glucan chains are capable of forming a triple-helix structure, and this tertiary structure may significantly influence their interaction with the helical coniotin A. Aib, an unnatural amino acid, strongly promotes helical conformations in peptides due to its high preference for α-helices, while simultaneously disrupting β-sheet formation.^59^ This property enhances the solubility and flexibility of peptide chains. Coniotin A contains six Aib residues enriched at each end of the peptide, along with four Iva residues, which collectively are predicted to induce a helical structure, enhancing the molecule’s bioactivity and metabolic stability. The identification of the BGC for coniotin A offers valuable tools for the biosynthesis of Aib-containing peptides, which are especially beneficial in drug design due to their enhanced stability and resistance to enzymatic degradation.

Besides the unique properties of the coniotin A peptide, the N-terminal MOTDA moiety plays a crucial role in its bioactivity. Several linear lipopeptides containing the same N-terminal polyketide moiety, such as SCH 666456, SCH 666457, and SCH 643432, have been identified as cell wall-active antifungals.^41, 92^ Using the corresponding highly reducing type I PKS as a probe, a series of fungal hybrid PKS-NRPS BGCs were identified and retrieved, presenting significant potential for discovering lipopeptides with diverse bioactivities. These discoveries could significantly advance antifungal agent development, particularly in generating drug leads against multidrug-resistant *C. auris*, a pathogen that efficiently colonizes the skin, contaminates the patient’s environment, facilitates rapid nosocomial transmission, and causes systemic infection outbreaks with mortality rates of 40–60%. Further studies on the crystallization of coniotin A and its interaction with β-glucan could elucidate the molecular details of its mechanism of action and aid in designing peptides with specific structural or functional properties targeting the fungal cell wall. As the fungal cell wall is absent in humans and exhibits a low potential for resistance development, it represents a promising therapeutic target for combating emerging fungal infections and drug resistance.

## Methods

### Cultivation and Fermentation conditions of Bacterial strains

*Streptomyces sp.* strains WAC1325, WAC1490, and WAC5858 were initially cultured on Mannitol Soya Flour (MS) agar (2% mannitol, 2% soya flour, 2% agar) at 30°C for 7 days to promote sporulation. A single colony from the sporulated MS agar culture was then transferred to Bennett’s agar plates and incubated under the same conditions for an additional 7 days for fermentation.

Bennett’s medium was prepared with the following composition per litre: 10 g potato starch, 2 g casamino acids, 1.8 g yeast extract, and 2 mL Czapek mineral mix. The Czapek mineral mix contained 10 g KCl, 10 g MgSO₄·7H₂O, 12 g NaNO₃, 0.2 g FeSO₄·7H₂O, 200 µL concentrated HCl, and was adjusted to 100 mL with double-distilled water (ddH₂O). The final pH was adjusted to 6.8, and the medium was autoclaved at 121°C for 45 minutes.

*Bacillus velezensis* WAC11084 strains were revived from cryopreserved stocks and cultured in Luria-Bertani (LB) broth or on LB agar plates, with incubation overnight at 37°C. For fermentation, the strain was transferred to Bennett’s medium and incubated at 37°C for 5 days.

### Culture Conditions for Fungal and Yeast Strains

*Coniochaeta hoffmannii* WAC11161, *Cryptococcus neoformans* strain H99, *Candida auris* (CBS10913, CBS12766, CBS12775, CBS12776), *Candida albicans* (ATCC 90028, ATCC 200955), *Candida parapsilosis* ATCC22019, *Candida tropicalis* ATCC200956, *Nakaseomyces glabratus*, *Saccharomyces cerevisiae* (BY4741, BY4742), and *Aspergillus fumigatus* (Af293, 1478) were cultured under standard eukaryotic conditions. Cultures were grown in YPD medium (1% yeast extract, 2% peptone, 2% dextrose) or Sabouraud Dextrose Broth (SDB, BD Difco) at 30–37°C. When required, strains were maintained on corresponding agar plates. To ensure cell viability, sterile techniques were employed, and cells were regularly passaged.

### Fermentation of Coniotins

To produce coniotin A, *C. hoffmannii* WAC11161 cultures were grown on Bennett’s agar plates and incubated for 8 days at 30°C.

### High-throughput cell-based screening of natural product library and prefractionated library

A high-throughput screening of a natural product library and a prefractionated library was performed to assess the advantages of fractionated libraries over crude extracts. The screening encompassed 379 crude extracts and 3,032 corresponding fractions, each tested in duplicate. The screening was performed against *C. albicans* ATCC 90028 and *C. auris* CBS 10913 using the Biomek Fxp Integrated Liquid Handler. *Candida* cultures were streaked on YPD agar for single colonies and incubated at 30°C for 48 hours. Cultures were then prepared to a final concentration of 10^3^ cells/mL in RPMI 1640 medium. In 384-well plates, 1 µL of crude extract, conditioned media, or fraction was mixed with 49 µL of the yeast culture using the Formulatrix Tempest Liquid Handler. Amphotericin B (8 µg/mL) served as a positive control. After 48 hours of incubation, plates were read at OD_530_ on a Biotek Neo microtiter plate reader. Data were normalized using control-based normalization, and hits were defined as wells exhibiting a minimum of 75% growth reduction for both *C. albicans* and *C. auris*.^93^

### Hit verification

Nine hits were selected for follow-up verification based on the initial screening data. The corresponding strains were revived from cryopreserved stocks, plated on Bennett’s agar, and incubated at 30°C for 7 days. The agar cultures were then crushed and extracted three times with methanol. Methanol extracts were pooled and evaporated using a rotary evaporator. The dry samples were resuspended in DMSO and loaded onto a prepacked C18 sample load cartridge. Fractionation was performed using a CombiFlash system (Teledyne ISCO, Inc.) equipped with REDISEP GOLD^®^ C18 reversed-phase columns. Separation was achieved at a flow rate of 12 mL/min using a water-acetonitrile (CH₃CN) gradient. A total of 24 fractions were collected per run and dried using Genevac Evaporators (Canadawide Scientific). The dried fractions were then dissolved in 200 µL DMSO with sonication, and their antifungal activity was evaluated against *Candida albicans* and *Candida auris*.

### Susceptibility test of antifungal agents

Minimum inhibitory concentration (MIC) determinations were performed following the National Committee for Clinical Laboratory Standards (NCCLS) protocol M27 (Reference Method for Broth Dilution Antifungal Susceptibility Testing of Yeasts). Several colonies from two-day-old cultures were resuspended in 0.85% saline to an initial OD_530_ of 0.11–0.14 and then diluted 1:2000 in RPMI 1640 medium. A two-fold serial dilution of test agents was prepared and added to the diluted culture in 96-well U-bottom plates. Column 11 served as the growth control (inoculum without drug), and Column 12 served as the sterile control (sterile media only). Both controls contained the same vehicle (e.g., DMSO) as the test wells. The sterile control readings were labelled “bkgd” (background), and the growth control readings were labelled “growth.” Growth inhibition was calculated as: % growth = [(OD_530_ − mean bkgd)/(mean growth - mean bkgd)]×100. For the bioactivity testing of fractions, 4 µL of DMSO-dissolved fractions were added to the diluted culture. After 48 hours of incubation at 30°C, optical density (OD) at 530 nm was measured using a BioTek Synergy Microplate Reader. MIC for fluconazole was defined as the lowest concentration that caused an 80% reduction in growth, while MIC for other drugs was set as the lowest concentration that completely inhibited growth.

### High-resolution mass spectrometry analysis

High-resolution mass spectrometry (HRMS) analyses of active fractions were performed using a qTOF LC/MS/MS system. An Agilent 1290 Infinity II LC System (Agilent Technologies) coupled with a qTOF 6550 mass detector was used to acquire mass spectra. The instrument operated in positive ionization mode with a capillary voltage of 3500 V, nozzle voltage 1000V, fragmentor 380. The dry gas flow rate was set to 14 L/min at 200°C with a nebulizer pressure of 35 psig. The sheath gas temperature was 350°C and sheath gas flow to 11 L/min. Data acquisition covered an *m/z* range of 100–3000 with a collection rate of 1spectra/sec.

Targeted MS/MS analysis performed on a list of specific precursor ions was used to confirm the structure of coniotin lipopepdides. The instrument settings were as follow: MS range was set up to 50–3000 m/z at a scan rate 1spectra/sec. The MS/MS range was set to 100–3000 m/z and at MS/MS scan rate 1 spectra/sec. The following fixed collision energies were used 20, 30, 40 and 50 eV.

Chromatographic separation was achieved using a gradient of H₂O (0.1% formic acid v/v) and acetonitrile (0.1% formic acid v/v) on an Eclipse SDB-C8 column (2.1 mm ID × 100 mm, 3.5 µm; Agilent, USA). The flow rate was 0.4 ml/min and the gradient started with 25%B for 0.5min, followed by a linear gradient to 100%B over 6.5min.

In the auto MS/MS method for GNPS analysis the mass range was set to 100–1700 m/z at a scan rate of 1 spectra/sec. The source parameters were as stated above. The isolation width was set to medium (4amu) with 3 fixed collision energies 10, 30, 60 eV. The chromatographic separation was performed using the same column and flow rate, but the pump method was different: from 0 to 2min 10%B, followed by a liner gradient to 100%B over 15 min.

### High-resolution mass spectrometry (HRMS) and chromatographic analysis

HRMS analyses of active fractions were performed on a qTOF LC/MS/MS system using an Agilent 1290 Infinity II LC System (Agilent Technologies) coupled with a qTOF 6550 mass detector. The instrument operated in positive ionization mode with the following settings: capillary voltage of 3500 V, nozzle voltage of 1000 V, and fragmentor voltage of 380 V. The dry gas flow rate was set to 14 L/min at 200°C, with a nebulizer pressure of 35 psig. Sheath gas temperature and flow were maintained at 350°C and 11 L/min, respectively. Data acquisition covered an *m/z* range of 100–3000 at a collection rate of 1 spectrum/sec.

Targeted MS/MS analysis was performed on specific precursor ions to confirm the structure of coniotin lipopeptides. MS was set to an *m/z* range of 50–3000 at a scan rate of 1 spectrum/sec. The MS/MS range was 100–3000 *m/z* with the same scan rate. Collision energies of 20, 30, 40, and 50 eV were applied for fragmentation.

Chromatographic separation was achieved using an Eclipse SDB-C8 column (2.1 mm ID × 100 mm, 3.5 µm; Agilent, USA) with a flow rate of 0.4 mL/min. The mobile phase comprised H₂O (0.1% formic acid, v/v) and acetonitrile (0.1% formic acid, v/v). The gradient started with 25% B for 0.5 min, followed by a linear increase to 100% B over 6.5 min.

For GNPS analysis, the mass range was set to 100–1700 *m/z* with a scan rate of 1 spectrum/sec. Source parameters were as stated above, and the isolation width was set to medium (4 amu) with fixed collision energies of 10, 30, and 60 eV. Chromatographic separation was performed on the same column with a flow rate of 0.4 mL/min. The gradient started at 10% B for 2 min, followed by a linear gradient to 100% B over 15 min.

### Identification of known antifungals by GNPS via HRMS/MS

Raw HRMS/MS data were converted to mzXML format and analyzed using the GNPS platform (Global Natural Product Social Molecular Networking, https://gnps.ucsd.edu) ^94^ via its online workflow (https://ccms-ucsd.github.io/GNPSDocumentation/). Data were filtered to exclude fragment ions within ± 17 Da of the precursor m/z, retaining the top six fragment ions within ± 50 Da throughout the spectrum. Mass tolerances were set to 1.0 Da for precursor ions and 0.5 Da for MS/MS fragment ions. A molecular network was generated with edges requiring a cosine score > 0.7 and at least six matched peaks. Nodes were connected only if they appeared in each other’s top 10 most similar nodes. Molecular families were capped at 100 nodes by removing the lowest-scoring edges. Spectra in the network were searched against GNPS spectral libraries using the same filtering criteria. Matches required a cosine score > 0.7 and at least six matched peaks. This approach enabled the identification of known antifungal compounds.

### Genome isolation

Genomic DNA was extracted from WAC1325, WAC1490, WAC5858, WAC11084, WAC11161, and WAC11175 for sequencing. Prokaryotic genomes were isolated from 48-hour Tryptic Soy Broth (TSB) cultures. Cells were harvested and treated with 1 mg/mL lysozyme, followed by 1% SDS and 0.5 mg/mL proteinase K at 55°C for 2 hours. Proteins were removed via chloroform extraction and centrifugation. DNA was precipitated using cold isopropanol, washed with 70% ethanol, and dissolved in TE buffer. Residual RNA was eliminated using 100 µg/mL RNase.

Fungal DNA was isolated using a CTAB-based method.^95^ Freeze-dried fungal cells were disrupted with glass beads and extracted with CTAB buffer (100 mM Tris-HCl, 0.7 M NaCl, 10 mM EDTA, 1% CTAB, 1% 2-mercaptoethanol, pH 7.5) at 65°C for 30 minutes. Proteins were removed by chloroform extraction and centrifugation. DNA was precipitated with isopropanol, washed with 70% ethanol, and dissolved in TE buffer.

### Genome sequencing and assembly

Genomic DNA was prepared for Illumina sequencing (MiSeq 2 × 300 bp reads) using the NEB Next Ultra V2 kit (New England Biosciences) with 500 ng of input DNA sonicated to 600 bp and size-selected with AMPure XP beads (Beckman Coulter). Sequencing was performed by the McMaster Genomics Facility, and reads were trimmed with Skewer v0.2.2 (-q 25, -Q25) and merged using FLASH v1.2.11.^96, 97^ De novo assembly was carried out with SPAdes v3.15.2 or SPAdes v3.15.4.^98^

For WAC11161, the draft genome assembly consisted of 210 contigs with a total length of 35.4 Mb and an N50 of 778,487. The 18S rRNA gene sequence, retrieved with RNAmmer v1.2, was identified via BLASTN searches.^99, 100^ The top hits were *Coniochaeta prunicola* (99.88% identity over 93% query coverage) and *Coniochaeta hoffmannii* (99.39% identity over 100% query coverage). Genome quality was assessed with BUSCO v5.4.7, confirming 97.9% completeness of conserved orthologs from the Sordariomycetes database (odb10).^101^ Illumina reads re-mapped using BWA MEM yielded 121X average coverage, a mean mapping quality of 59.89, and a mean base quality of Q33.89 (> 99.95% base accuracy).^96, 102^ Breseq v0.37.0 showed 99.0% of reads mapped to the assembly.^102^ Fungismash (antiSMASH 7.0^84^) identified a 181,089 bp biosynthetic gene cluster within a 444,462 bp contig associated with coniotin A. These results demonstrate a high-quality draft assembly of the *Coniochaeta* genome with excellent coverage and completeness, enabling further analysis.

### Purification of active compound 1 from WAC11161

Single colonies of *C. hoffmannii* WAC11161 were picked from YPD agar plates after two days of growth and incubated on 20 Bennett’s agar plates (30 × 42 × 3 cm, 500 mL/plate) at 30°C for 8 days. The fermented agar was blended and extracted with an equivalent volume of methanol under shaking. The methanol extract was concentrated under reduced pressure and resolubilized in 200 mL of methanol. Following centrifugation, the crude supernatant was combined with 5 g of C18 resin, dried by rotary evaporation, and loaded onto a RediSep C18 Gold column (86 g) for purification using a CombiFlash system (Teledyne ISCO, Inc.) at a flow rate of 66 mL/min. Partially purified compound 1 was eluted with a linear gradient of 10–100% acetonitrile (0.1% formic acid). Active fractions were identified through bioactivity testing and LC-MS analysis, pooled, lyophilized, and further purified on an LH20 column (3 × 40 cm) with methanol as the eluent (fraction size: 10 mL).

Bioactive fractions (fractions 7–11) were concentrated to dryness using Genevac Evaporators (Canadawide Scientific) and subjected to HPLC purification (1260 Agilent Technologies) on an Eclipse SDB-C8 column (4.6 × 250 mm, 5 µm). The compound was eluted with 70% acetonitrile (0.1% formic acid) and assessed by HR-ESI-MS in positive ion mode. Compound 1: calculated mass for C_98_H_170_N_21_O_26_ [M + H]^+^: 2057.2620; observed 2057.2614. Approximately 10 mg of compound 1 was obtained. NMR data were acquired on a Bruker AVIII 700 MHz instrument equipped with a cryoprobe.

### Chemical hydrolysis

Acid hydrolysis was performed as previously described.^48^ Briefly, 1 mg of the product was resuspended in 500 µL of 6N HCl and incubated at 100°C for 20 hours. After the reaction, 1 mL of ethyl acetate was added to the mixture. The organic phase was separated, dried, and analyzed, while the aqueous phase was concentrated under nitrogen and subjected to chemical modification with Marfey’s reagent.

Partial hydrolysis was conducted similarly using 3M HCl at 90°C for 5 hours. Aliquots were taken at different time points and analyzed by HR-LC-MS. Selected cleaved peptides were further characterized using targeted MS/MS analysis.

### Marfey’s reagent chemical modification

Marfey’s reagent chemical modification was performed as described previously.^48^ Briefly, approximately 0.2 mg of each amino acid standard was dissolved in 50 µL of H₂O, followed by the addition of 20 µL 1 M NaHCO₃ and 100 µL of 1% Marfey’s reagent (Nα-(2,4-dinitro-5-fluorophenyl)-L-alaninamide, Acros Organics) in acetone. The mixtures were agitated at 40°C for 1 hour, and the reactions were stopped by adding 10 µL of 2N HCl. Reaction products were dried under nitrogen, dissolved in ~ 1.7 mL methanol, and individually injected (0.5 µL) into a UPLC-MS for analysis.

The digested and derivatized peptaibiotics were generated using the following procedure: approximately 0.2–0.3 mg of compounds 1–3 were separately hydrolyzed in 500 µL of 6N HCl at 90°C for 24 hours. The hydrolysates were dried under nitrogen and treated with 25 µL H₂O, 25 µL 1 M NaHCO₃, and 50 µL of 1% Marfey’s reagent in acetone. Reactions were agitated at 40°C for 1 hour and stopped with 5 µL of 2N HCl. The products were dried under nitrogen, dissolved in ~ 200 µL methanol, and injected into the UPLC-MS under the same conditions as the standards.

### Papain hydrolysis

Compound 1 (1 mg dissolved in 10 µL DMSO) was hydrolyzed in a reaction mixture containing 200 µL of 0.05 M Tris-HCl buffer (pH 6.8), 20 mM 2-mercaptoethanol, 0.5 mM EDTA, and 7 mg of papain, as previously described.^103^ The reaction was incubated at 37°C with shaking for 4 days to ensure complete hydrolysis. Hydrolysis progress was monitored daily using LC-MS. Aliquots (20 µL) were taken from the reaction mixture, and the supernatant was removed. The pellet was resuspended in 20 µL methanol, and 5 µL of the resuspension was analyzed by LC-MS. Prominent peptide molecular ions were selected for further fragmentation analysis.

### Hemolysis Testing

Human blood collected in K2-EDTA tubes was obtained from BioIVT (New York, USA). The blood was centrifuged at 500 × g for 5 minutes, and the plasma was removed. Red blood cells (RBCs) were washed twice with 150 mM NaCl in a volume equal to the removed plasma. After the second wash, RBCs were resuspended in phosphate-buffered saline (PBS, pH 7.4) at a volume equivalent to the plasma to maintain hematocrit levels. Compound solutions (1 µL) were added to 96-well V-bottom plates using a Labcyte Echo acoustic dispenser (Beckman Coulter). DMSO was included at a constant 1% (v/v) final concentration, with DMSO-only controls as negative controls. Triton X-100 (10 µL, starting at 20% and serially diluted 2-fold to 0.02%) served as a positive control. RBCs were diluted 1:50 in PBS (pH 7.4), and 99 µL of this suspension was added to each well. Plates were incubated at 37°C for 1 hour, followed by centrifugation at 500 × g for 5 minutes to pellet intact RBCs. A 65 µL aliquot of the supernatant was transferred to a clear, flat-bottom 96-well plate, and absorbance was measured at 540 nm. Coniotin A and B were tested at a starting concentration of 128 µg/mL, while Iturin A, Caspofungin, and Amphotericin B were tested starting at 256 µg/mL. Compounds were diluted 2-fold to create an 11-point dose-response curve. Each concentration was tested in duplicate.

### Cytotoxicity Testing

On Day 1, HEK293 cells (ATCC CRL-1573; generation 6) were seeded at 7500 cells/well in 384-well tissue culture-treated white plates with 50 µL of Dulbecco Modified Eagle Medium (DMEM) supplemented with 10% fetal bovine serum (FBS), 2 mM L-glutamine, 100 units/mL penicillin, and 100 µg/mL streptomycin. Cells were incubated for 18 hours at 37°C under 5% CO₂. On Day 2, 500 nL of compound solutions and DMSO were added to the wells using a Labcyte Echo acoustic dispenser (Beckman Coulter) and a Combi nL dispenser (ThermoFisher), maintaining a final DMSO concentration of 1% across all wells. After 48 hours of incubation, cell viability was assessed using Promega CellTiter-Glo 2.0 reagent (Fisher Scientific). A total of 50 µL of CellTiter-Glo was added directly to each well, plates were shaken for 2 minutes, and then incubated for 10 minutes at room temperature. Luminescence was measured on a Neo2 plate reader (Biotek) using a luminescence fiber optic. Untreated cells and DMSO-only treated cells were used as controls. Compounds were tested in triplicate at each concentration. Coniotin A and B were tested starting at 128 µg/mL, while Iturin A, Caspofungin, and Amphotericin B were tested starting at 256 µg/mL. Compounds were serially diluted 2-fold to generate an 11-point dose-response curve.

Dose-response curves were fitted using a four-parameter logistic (4PL) non-linear regression model, constrained to a minimum response of 0 and a maximum response of 1. The 4PL equation used was:

$$\text{y}=\text{d}+\left(\text{a}-\text{d}\right)/{\left(1+\text{x}/\text{c}\right)}^{\text{b}}$$

Where

y = the sample response in relative luminescence units

x = the drug concentration

a = the maximum response for infinite standard concentration

b = -Hill slope

c = inflection point

d = the response at a standard concentration of 0

#### Rapid C. elegans – C. albicans Antifungal Activity Assay

The *C. elegans glp-4(bn2)*; *sek-1(km4)* double mutant was used for a rapid co-infection antifungal assay, as previously described.^104^ Briefly, 70 µL of screening medium (30% BHI in M9 buffer containing 90 µg/mL kanamycin, 200 µg/mL ampicillin, and 200 µg/mL streptomycin), 450 nL of test compounds or DMSO vehicle, 15 worms, and 10 µL of *C. albicans* ATCC90028 (2.5 × 10⁴ cells/mL in PBS) were added to 96-well clear flat-bottom plates. The Union Biometrica COPAS-BIOSORT was used to dispense worms, and the plates were sealed with a porous film. The assay plates were incubated at 25°C for 96 hours before imaging with a Nikon Multizoom AZ100M microscope equipped with a 2X Plan Fluor objective. Images were captured using NIS-Elements AR software (v5.11, Nikon).

#### C. elegans survival assay

A synchronized population of the *C. elegans* double mutant strain AU37 (*glp-4(bn2)*; *sek-1(km4)*) was grown on nematode growth medium (NGM) at 25°C for 48 hours prior to infection. The *C. auris* infection protocol was adapted from a previously described method and scaled for 96-well plates.^105^ Briefly, worms were washed with M9 buffer and placed onto brain heart infusion (BHI) agar plates supplemented with 50 µg/mL kanamycin and seeded with a *C. auris* lawn. Worms were allowed to feed on the lawn for 3 hours before being washed off and transferred to empty NGM plates to crawl for 1 hour. Using the Union Biometrica COPAS-BIOSORT, 25 worms were dispensed into each test well of a 96-well plate. The media was adjusted to a final composition of 20% BHI and 80% M9 buffer, supplemented with 10 µg/mL cholesterol. Test conditions included DMSO vehicle, 1× MIC of coniotin A, and amphotericin B, each tested in triplicate. Plates were covered with a porous film, incubated at 25°C, and worm survival was monitored every 8 hours for 48 hours.

### HIP screening

*C. albicans* haploinsufficiency (HIP) was performed as previously described.^63^ Glycerol stock pools of heterozygous (HET) double-barcoded deletion mutants were thawed, diluted to an OD_600_ of 0.05 into a 60 mL YPD culture, and grown at 30°C under shaking conditions for 1.5 hours. Subsequently, 1 mL of the sub-cultured HET pool was aliquoted into triplicate culture tubes, each containing 1mL YPD medium with coniotin A or a DMSO solvent control. These cultures were grown at 30°C under shaking conditions for 18 hours. Cells were pelleted by centrifugation, the supernatant was removed, and cell pellets were stored at −80°C. Cell pellets were digested with Zymolase in buffer (1 M sorbitol, 10 mM sodium EDTA, 14 mM β-mercaptoethanol, 15 units of Zymolase enzyme) prior to genomic DNA extraction using the PureLink Genomic DNA Extraction kit, as per the manufacturer’s instructions (Invitrogen). Genomic DNA was recovered from columns provided by the kit using 10 mM Tris-HCl pH 8.0 and quantified using the PicoGreen DNA quantification kit (Invitrogen). Barcodes were PCR amplified with Takara Ex-Taq (Clonetech) using 150 ng of genomic DNA. UP-TAG primers (UP-TAG U and UP-TAG INX) and DOWN-TAG primers (DOWN-TAG U and DOWN-TAG INX) were used.^106^ Equal quantities of UP-TAG and DOWN-TAG pools were combined to form a sequencing library, which was sequenced on an Illumina Next-Seq500 instrument (Mid-Output, V2 Chemistry) using specific primers to sequence and index the UP- (UP-TAG S and UP-TAG SINX) and DOWN-TAG (DOWN-TAG S and DOWN-TAG SINX) pools for each sample.^106^ Barcode-sequence reads were mapped to an artificial genome containing known UP-TAG and DOWN-TAG sequences of each strain and compiled for each indexed sample. If a specific UP-TAG or DOWN-TAG had more than one of its triplicate samples in the solvent control condition with read counts < 20% of the median read per million mapped, these reads were filtered and omitted from further analysis. Log_2_ fold differences for each strain’s UP-TAG and DOWN-TAG were calculated.

### Intracellular drug accumulation assay

The intracellular drug accumulation assay was performed in triplicate using caspofungin, iturin A, and coniotin A, following a previously described protocol.^64^ *C. albicans* ATCC90028 and *C. auris* CBS10913 were used for the experiments. Overnight cultures (OD_530_ = 1.6–1.8) were subcultured into fresh SDB and grown at 30°C with shaking until reaching an OD_530_ of 0.6. Cells were pelleted, washed twice with PBS, and resuspended in 15 mL fresh PBS. Aliquots (875 µL) were transferred into ten 1.5 mL Eppendorf tubes, resulting in a final concentration of ~ 3.3 × 10^7^ cells/mL. Samples were equilibrated at 30°C for 5 minutes before treatment with test agents at a final concentration of 20 µM for 10 minutes. After incubation, 800 µL of the culture was layered onto 700 µL of pre-cooled silicone oil (9:1 mixture of silicone oil AR200 and Sigma High-Temperature silicone oil) with 13.3% hexane and centrifuged at 13,000 × g to pellet cells through the oil. The supernatant and oil layers were carefully removed by pipetting. Pellets were transferred to new tubes, washed twice with water, and extracted with 150 µL DMSO/MeOH (2:1). Extracts were analyzed and quantified using HR-ESI-MS on an Agilent 1290 Infinity II HPLC system coupled with a qTOF 6550 ESI/MS, equipped with an Eclipse SDB-C8 column (2.1 mm ID × 100 mm, 3.5 µm; Agilent, USA) and operated in positive ion mode. The mobile phase consisted of 0.1% formic acid in water (phase A) and 0.1% formic acid in acetonitrile (phase B), at a flow rate of 0.3 mL/min. Error bars represent the standard error of the mean of three biological replicates. All compounds used in biological assays were of ≥ 95% purity.

### Calcofluor white staining and widefield microscopy

Fresh overnight cultures of *C. albicans* ATCC90028, *C. neoformans* H99, and *C. auris* CBS10913 were sub-cultured in YPD broth to an OD_600_ of 0.1. Cultures were treated with DMSO or half the MIC of test agents and incubated at 30°C with shaking for 4 hours. Cells were washed with PBS, resuspended in PBS to an OD_600_ of 3, and stained with calcofluor white (10 µg/mL). Imaging was performed using a Nikon Eclipse Ti inverted microscope equipped with a 100× Plan Fluor Apo λ oil immersion objective. Micrographs were captured as raw 16-bit TIFF files using NIS-Elements AR software (v4.50, Nikon) with a 4′,6-diamidino-2-phenylindole (DAPI) hybrid filter, and a SpectraX LED fluorescence source (20% power). Probe exposure was set to 50 ms, with gain at 0, and minimum/maximum values held constant across all samples. Fluorophore Intensity Quantification: Image analysis was performed using ImageJ and CellProfiler.^107^ An ImageJ macro was used to subtract background with a rolling ball radius of 50 pixels. The processed images were analyzed in CellProfiler (v4.2.1) to identify cells as primary objects and quantify fluorescence intensities for whole cells and cell edges. Calcofluor intensity per cell was calculated by dividing the total fluorescence intensity by the number of cells.

### Concanavalin A (Alexa Fluor^™^ 647 conjugate) staining and confocal microscopy

Cell surface properties were analyzed using Alexa Fluor 647-ConA (Invitrogen C21421) staining as previously described.^74^ An overnight culture of *Candida albicans* ATCC 90028 was subcultured to an initial OD₆₀₀ of 0.1 and incubated at 30°C with shaking for 4 hours in the presence of half the MIC of coniotin A, caspofungin, or an equivalent volume of DMSO. Treated cells were collected by centrifugation, washed twice with 1 mL PBS, and briefly sonicated to remove debris. The cells were then stained with 50 µg/mL Alexa Fluor 647-ConA for 10 minutes at room temperature. After staining, the cells were washed three times with PBS, resuspended to an OD₆₀₀ of 1, and mounted on square coverslips (#1.5, 12-541-AP, Fisherbrand) using ProLong^™^ Diamond Antifade Mountant (P36961, Invitrogen). Imaging was performed with a Zeiss LSM 980 Inverted Confocal Microscope (Zeiss Axio Observer.Z1/7 stand), equipped with an Airyscan 2 detector and a 63x/1.4 oil-immersion objective. The sample was excited using a 639 nm laser and emission was detected using a 528/29 + 697/38 nm multi-band-pass filter. Z-stack images, composed of 40–60 slices with a step size of 0.2 µm, were acquired in Airyscan mode using a pixel size of 56 nm. 3D Airyscan processing was performed in Zen (Carl Zeiss, Germany) to generate the super-resolution image. Cell perimeter quantification was carried out using Fiji software.^108^

### Glucan pull-down assay

A glucan pull-down assay was conducted to investigate the interaction between Coniotin A and polysaccharides. β-Glucan (1 mg/mL; Millipore Sigma 346210) and chitin (1 mg/mL; Sigma C9752) were suspended in 1× PBS (pH 7.0). Coniotin A (2 µL, 3.2 mg/mL stock solution) was added to 200 µL of each suspension, followed by incubation with shaking at 30°C for 1 hour. After incubation, samples were centrifuged for 5 minutes to separate the supernatant and pellet fractions. The supernatant was collected to analyze unbound coniotin A. The polysaccharide pellets were washed three times with PBS and extracted with DMSO using sonication to release bound Coniotin A. Coniotin A was identified and quantified in both the supernatant and DMSO extracts using an Agilent 1290 Infinity II LC system (Agilent Technologies) coupled with a qTOF 6550 mass detector and an Eclipse SDB-C8 column (2.1 mm ID × 100 mm, 3.5 µm; Agilent, USA). The quantification of coniotin A was based on MS peak area and compared to pure coniotin A and caspofungin (Merck) standards. All experiments were performed in triplicate, and results are presented as mean ± standard deviation (SD).

### Synthesis and application of coniotin A-BODIPY

Coniotin A (2 mg, 0.97 µmol, 1 eq) was dissolved in DMSO, and a solution of Bodipy-FL-ethylenediamine (3.6 µg, 9.7 µmol, 10 eq) in DMSO was added. Benzotriazol-1-yloxytripyrrolidinophosphonium hexafluorophosphate (PyBop) (10 µL of 1M solution in 50% DMF/DMSO, excess) and N-methylmorpholine (1 µL of 1M solution in 50% DMF/DMSO, excess) were then added to the mixture. The reaction was carried out at room temperature (RT) with stirring for 40 minutes, after which the reaction mixture was lyophilized. The product was purified by semi-preparative HPLC (1260 Agilent Technologies) using an Eclipse SDB-C8 reverse-phase column (4.6 × 250 mm, 5 µm) with a gradient elution of water (0.1% formic acid) and acetonitrile (0.1% formic acid). The flow rate was set to 2 mL/min, and the compound was eluted with 80% acetonitrile (0.1% formic acid) and assessed by HR-ESI-MS in positive ion mode. Calculated mass for C_130_H_208_B_2_F_4_N_29_O_26_ [M + 1H]^2+^ 1344.7981; observed: 1344.7984. Purified product was collected, lyophilized, and stored for subsequent use.

β−1,3-glucan particles (1 mg/mL) were incubated with 120 µM BODIPY-conjugated coniotin A in PBS for 1 hour. After incubation, the particles were washed three times with PBS and imaged using a Zeiss LSM 980 Upright Confocal Microscope (Zeiss Axio Imager.Z2 stand) with a GaAsp-Pmt3 detector using 63x/1.4 oil-immersion objective. BODIPY was excited with a 488 nm laser, and fluorescence was detected using a FITC channel (525/28 nm bandpass). To observe the general morphology of the glucan particles, a transmitted confocal laser image was captured in brightfield mode. Merged images were then generated by overlaying the brightfield and fluorescence signals in ImageJ.

### Glucanase Digestion Assay

A glucanase digestion assay was performed using laminarin as the substrate to evaluate the effects of coniotin A on β-(1,3)-D-glucanase activity. Laminarin (125 µg/mL, Sigma L9634) was digested with β-(1,3)-D-glucanase (250 µg/mL, Sigma 67138) in 100 µL of 1× PBS (pH 7.0) containing 31.2, 62.4, or 124.8 µM coniotin A, or an equivalent volume of DMSO as a vehicle control. Reactions were incubated at 30°C for 30 minutes. A negative control was performed by omitting β-(1,3)-D-glucanase. The reactions were quenched by adding 100 µL methanol, and 5 µL of the resulting mixture was analyzed using an Agilent 1290 Infinity II LC System (Agilent Technologies) coupled with a qTOF 6550 mass detector on a Luna HILIC 200 Å column (4.5 mm ID x 100 mm ,5µm; Phenomenex). The oligosaccharide product (hexa-glucose) generated from the digestion of laminarin was quantified. Data were expressed as mean ± standard deviation (SD) from three independent experiments.

### Kinetic assay of β-glucan activation of Limulus coagulation factor G

The kinetic chromogenic assay specific to (1,3)-β-D-glucan was conducted using the Glucatell^®^ (1,3)-Beta-D-Glucan Detection Reagent Kit (Associates of Cape Cod) following the manufacturer’s instructions. A glucan standard solution (100 pg/mL) was prepared by dissolving the supplied glucan standard in LAL Reagent Water. The Glucatell reagent was reconstituted with 2.8 mL of LAL Reagent Water and 2.8 mL of Pyrosol buffer. The mixture was gently swirled until fully dissolved and used within 10 minutes. A 100 pg/mL (1,3)-β-D-glucan solution was incubated with coniotin A (CNA) at concentrations of 0.625 µg/mL, 5 µg/mL, and 40 µg/mL, or with an equivalent volume of DMSO as a control, at 30°C for 1 hour. Subsequently, 25 µL of each sample was added to designated wells, followed by 100 µL of reconstituted Glucatell reagent. LAL Reagent Water served as a negative control. All samples and controls were assayed in triplicate. The plate was placed in a preheated plate reader at 37°C, shaken briefly, and read at 405 nm with a Biotek Neo plate reader. Kinetic readings were recorded every 30 seconds over 1 hour. Results were presented as mean ± SD from triplicate experiments.

### Aniline blue staining and confocal microscopy

The visualization of (1,3)-β-glucan was performed using aniline blue staining, as previously described.^74, 109^ Overnight cultures of *Candida albicans* ATCC 90028 were subcultured into YPD at an OD₆₀₀ of 0.1 and incubated with shaking at 30°C for 6 hours in the presence of half MIC of coniotin A or an equivalent volume of DMSO. Following treatment, cells were washed three times with PBS, resuspended to an OD₆₀₀ of 2, and stained with aniline blue (100 µg/mL) for 20 minutes. After staining, the cells were washed three times with PBS, resuspended in PBS to an OD₆₀₀ of 0.8, and mounted on square coverslips (#1.5, 12-541-AP, Fisherbrand) for imaging. Confocal fluorescence microscopy was performed using a Zeiss LSM 980 Upright Confocal Microscope (Zeiss Axio Imager.Z2 stand) with a GaAsp-Pmt3 detector using 63x/1.4 oil-immersion objective. Aniline blue was excited using the 405 nm laser and fluorescence was collected in the full visible spectrum (585/172 nm bandpass) to assess (1,3)-β-glucan distribution on the cell surface. Images were captured with consistent excitation power, detector gain, scanning speed and pixel size settings for comparability. β−1,3-glucan particles (Millipore Sigma 346210) were stained with aniline blue and visualized following the same protocol.

### Transmission Electron Microscopy

To investigate morphological alterations, *C. auris* CBS12766 and *C. neoformans* H99 were cultured in RPMI 1640 medium containing half MIC of coniotin A, amphotericin B, or an equivalent volume of DMSO as a vehicle control until detectable growth was observed. Cells were harvested by centrifugation and resuspended in a fixative solution containing 4% paraformaldehyde (PFA) and 2.5% glutaraldehyde in 0.1 M phosphate buffer (pH 7.4) with 1% Triton X-100. The samples were fixed for 15 minutes at room temperature and stored overnight at 4°C. Samples were prepared for TEM as previously described^110, 111^ with ethanol (EtOH) used for dehydration instead of acetone. Ultrathin sections were prepared using a Leica UCT ultramicrotome, mounted onto copper grids, and post-stained with uranyl acetate and lead citrate. Sections were visualized using a JEOL JEM 1200 EX TEMSCAN transmission electron microscope (JEOL, Peabody, MA, USA) operating at 80 kV. Images were acquired with an AMT 4-megapixel digital camera (Advanced Microscopy Techniques, Woburn, MA, USA).

For the visualization of *C. elegans – C. auris* infection, *C. elegans* infected with *C. auris* for 20 hours were fixed in a buffer containing 3.2% formaldehyde and 0.2% glutaraldehyde in 0.15 M sodium cacodylate buffer (pH 7.2) for 4 hours at room temperature. Sample preparation followed the same protocol as described above.^112^

## Supplementary Material

Supplement 1 Table 1 is available in the Supplementary Files section.

## Data Availability

The Whole Genome Shotgun (WGS) sequencing data for WAC1325, WAC1490, WAC58558, WAC11084, WAC11161, and WAC11175 have been deposited in GenBank under the BioProject accession number PRJNA1171131. For review, the data can be accessed at https://submit.ncbi.nlm.nih.gov/subs/wgs_batch/SUB14773833/. Data will be publicly available as of the publication date. Further inquiries and resource requests should be directed to the Lead Correspondent, Gerard D. Wright (wrightge@mcmaster.ca), and will be provided upon reasonable request.

## References

[1] Iliev I. D. Focus on fungi. Cell 187, 5121–5127 (2024). 39303681 10.1016/j.cell.2024.08.016 PMC11722117

[2] Denning D. W. Global incidence and mortality of severe fungal disease. Lancet Infect. Dis. 24, e428–e438 (2024). 38224705 10.1016/S1473-3099(23)00692-8

[3] Brown G. D. Hidden Killers: Human Fungal Infections. Sci. Transl. Med. 4, 165rv13–165rv13 (2012). 10.1126/scitranslmed.3004404 23253612

[4] Pfaller M. A. & Diekema D. J. Epidemiology of Invasive Candidiasis: a Persistent Public Health Problem. Clin. Microbiol. Rev. 20, 133–163 (2007). 17223626 10.1128/CMR.00029-06 PMC1797637

[5] Fisher M. C., Hawkins N. J., Sanglard D. & Gurr S. J. Worldwide emergence of resistance to antifungal drugs challenges human health and food security. Science 360, 739–742 (2018). 29773744 10.1126/science.aap7999

[6] Lyman M. Worsening Spread of Candida auris in the United States, 2019 to 2021. Ann. Intern. Med. 176, 489–495 (2023). 36940442 10.7326/M22-3469 PMC11307313

[7] Ruiz-Gaitán A. An outbreak due to Candida auris with prolonged colonisation and candidaemia in a tertiary care European hospital. Mycoses 61, 498–505 (2018). 29655180 10.1111/myc.12781

[8] WHO fungal priority pathogens list to guide research, development and public health action. https://www.who.int/publications/i/item/9789240060241.

[9] Kadri S. S. Key Takeaways From the U.S. CDC’s 2019 Antibiotic Resistance Threats Report for Frontline Providers. Crit. Care Med. 48, 939 (2020). 32282351 10.1097/CCM.0000000000004371 PMC7176261

[10] Sabino R., Veríssimo C., Pereira Á. A. & Antunes F. Candida auris, an Agent of Hospital-Associated Outbreaks: Which Challenging Issues Do We Need to Have in Mind? Microorganisms 8, E181 (2020). 10.3390/microorganisms8020181 PMC7074697 32012865

[11] Lockhart S. R. Simultaneous Emergence of Multidrug-Resistant Candida auris on 3 Continents Confirmed by Whole-Genome Sequencing and Epidemiological Analyses. Clin. Infect. Dis. Off. Publ. Infect. Dis. Soc. Am. 64, 134 (2016). 10.1093/cid/ciw691 PMC5215215 27988485

[12] Newman D. J. & Cragg G. M. Natural Products as Sources of New Drugs over the Nearly Four Decades from 01/1981 to 09/2019. J. Nat. Prod. 83, 770–803 (2020). 32162523 10.1021/acs.jnatprod.9b01285

[13] Wright G. D. Something old, something new: revisiting natural products in antibiotic drug discovery. Can. J. Microbiol. 60, 147–154 (2014). 24588388 10.1139/cjm-2014-0063

[14] Aminov R. I. A Brief History of the Antibiotic Era: Lessons Learned and Challenges for the Future. Front. Microbiol. 1, 134 (2010). 21687759 10.3389/fmicb.2010.00134 PMC3109405

[15] Harvey A. L., Edrada-Ebel R. & Quinn R. J. The re-emergence of natural products for drug discovery in the genomics era. Nat. Rev. Drug Discov. 14, 111–129 (2015). 25614221 10.1038/nrd4510

[16] Walsh C. T. A chemocentric view of the natural product inventory. Nat. Chem. Biol. 11, 620–624 (2015). 26284660 10.1038/nchembio.1894

[17] Pye C. R., Bertin M. J., Lokey R. S., Gerwick W. H. & Linington R. G. Retrospective analysis of natural products provides insights for future discovery trends. Proc. Natl. Acad. Sci. U. S. A. 114, 5601–5606 (2017). 28461474 10.1073/pnas.1614680114 PMC5465889

[18] Shendure J. & Ji H. Next-generation DNA sequencing. Nat. Biotechnol. 26, 1135–1145 (2008). 18846087 10.1038/nbt1486

[19] Wang Y., Zhao Y., Bollas A., Wang Y. & Au K. F. Nanopore sequencing technology, bioinformatics and applications. Nat. Biotechnol. 39, 1348–1365 (2021). 34750572 10.1038/s41587-021-01108-x PMC8988251

[20] Cimermancic P. Insights into secondary metabolism from a global analysis of prokaryotic biosynthetic gene clusters. Cell 158, 412–421 (2014). 25036635 10.1016/j.cell.2014.06.034 PMC4123684

[21] Kang H.-S. & Brady S. F. Arimetamycin A: improving clinically relevant families of natural products through sequence-guided screening of soil metagenomes. Angew. Chem. Int. Ed Engl. 52, 11063–11067 (2013). 24038656 10.1002/anie.201305109 PMC3878715

[22] Yamanaka K. Direct cloning and refactoring of a silent lipopeptide biosynthetic gene cluster yields the antibiotic taromycin A. Proc. Natl. Acad. Sci. U. S. A. 111, 1957–1962 (2014). 24449899 10.1073/pnas.1319584111 PMC3918841

[23] Culp E. J. Evolution-guided discovery of antibiotics that inhibit peptidoglycan remodelling. Nature 578, 582–587 (2020). 32051588 10.1038/s41586-020-1990-9

[24] Thornburg C. C. NCI Program for Natural Product Discovery: A Publicly-Accessible Library of Natural Product Fractions for High-Throughput Screening. ACS Chem. Biol. 13, 2484–2497 (2018). 29812901 10.1021/acschembio.8b00389 PMC8130845

[25] Appleton D. R., Buss A. D. & Butler M. S. A Simple Method for High-Throughput Extract Prefractionation for Biological Screening. Chim. Int. J. Chem. 61, 327–331 (2007).

[26] Wagenaar M. M. Pre-fractionated microbial samples–the second generation natural products library at Wyeth. Mol. Basel Switz. 13, 1406–1426 (2008). 10.3390/molecules13061406 PMC6245344 18596666

[27] Grkovic T. National Cancer Institute (NCI) Program for Natural Products Discovery: Rapid Isolation and Identification of Biologically Active Natural Products from the NCI Prefractionated Library. ACS Chem. Biol. 15, 1104–1114 (2020). 32223208 10.1021/acschembio.0c00139 PMC7171602

[28] Cook M. A. Lessons from assembling a microbial natural product and pre-fractionated extract library in an academic laboratory. J. Ind. Microbiol. Biotechnol. 50, kuad042 (2023). 38052426 10.1093/jimb/kuad042 PMC10724011

[29] Petras D. GNPS Dashboard: collaborative exploration of mass spectrometry data in the web browser. Nat. Methods 19, 134–136 (2022). 34862502 10.1038/s41592-021-01339-5 PMC8831450

[30] Aron A. T. Reproducible molecular networking of untargeted mass spectrometry data using GNPS. Nat. Protoc. 15, 1954–1991 (2020). 32405051 10.1038/s41596-020-0317-5

[31] Sy-Cordero A. A., Pearce C. J. & Oberlies N. H. Revisiting the enniatins: a review of their isolation, biosynthesis, structure determination, and biological activities. J. Antibiot. (Tokyo) 65, 541–549 (2012). 22990381 10.1038/ja.2012.71 PMC3573854

[32] Haese A., Schubert M., Herrmann M. & Zocher R. Molecular characterization of the enniatin synthetase gene encoding a multifunctional enzyme catalysing N-methyldepsipeptide formation in Fusarium scirpi. Mol. Microbiol. 7, 905–914 (1993). 8483420 10.1111/j.1365-2958.1993.tb01181.x

[33] Hornbogen T. Functional Characterization of the Recombinant N-Methyltransferase Domain from the Multienzyme Enniatin Synthetase. ChemBioChem 8, 1048–1054 (2007). 17471480 10.1002/cbic.200700076

[34] Jakab Á. Physiological and transcriptional profiling of surfactin exerted antifungal effect against Candida albicans. Biomed. Pharmacother. 152, 113220 (2022). 35671583 10.1016/j.biopha.2022.113220

[35] Koglin A. Structural basis for the selectivity of the external thioesterase of the surfactin synthetase. Nature 454, 907–911 (2008). 18704089 10.1038/nature07161 PMC2854587

[36] Takatsuki A., Arima K. & Tamura G. TUNICAMYCIN, A NEW ANTIBIOTIC. I ISOLATION AND CHARACTERIZATION OF TUNICAMYCIN. J. Antibiot. (Tokyo) 24, 215–223 (1971). 5572750 10.7164/antibiotics.24.215

[37] Travers K. J. Functional and Genomic Analyses Reveal an Essential Coordination between the Unfolded Protein Response and ER-Associated Degradation. Cell 101, 249–258 (2000). 10847680 10.1016/s0092-8674(00)80835-1

[38] Wyszynski F. J., Hesketh A. R., Bibb M. J. & Davis B. G. Dissecting tunicamycin biosynthesis by genome mining: cloning and heterologous expression of a minimal gene cluster. Chem. Sci. 1, 581–589 (2010).

[39] Tsvetanova B. C., Kiemle D. J. & Price N. P. J. Biosynthesis of tunicamycin and metabolic origin of the 11-carbon dialdose sugar, tunicamine. J. Biol. Chem. 277, 35289–35296 (2002). 12093793 10.1074/jbc.M201345200

[40] Hu Y. Identification and Proposed Relative and Absolute Configurations of Niphimycins C–E from the Marine-Derived Streptomyces sp. IMB7–145 by Genomic Analysis. J. Nat. Prod. 81, 178–187 (2018). 29308897 10.1021/acs.jnatprod.7b00859

[41] Hegde V. R. Novel Fungal Metabolites as Cell Wall Active Antifungals Fermentation, Isolation, Physico-chemical Properties, Structure and Biological Activity. J. Antibiot. (Tokyo) 56, 437–447 (2003). 12870808 10.7164/antibiotics.56.437

[42] Johnson A. R. & Carlson E. E. Collision-Induced Dissociation Mass Spectrometry: A Powerful Tool for Natural Product Structure Elucidation. Anal. Chem. 87, 10668–10678 (2015). 26132379 10.1021/acs.analchem.5b01543

[43] Degenkolb T., Berg A., Gams W., Schlegel B. & Gräfe U. The occurrence of peptaibols and structurally related peptaibiotics in fungi and their mass spectrometric identification via diagnostic fragment ions. J. Pept. Sci. 9, 666–678 (2003). 14658788 10.1002/psc.497

[44] Heinze S. Lipohexin, a New Inhibitor of Prolyl Endopeptidase from Moeszia lindtneri (HKI-0054) and Paecilomyces sp. (HKI-0055; HKI-0096). I. Screening, Isolation and Structure Elucidation. J. Antibiot. (Tokyo) 50, 379–383 (1997). 9207906 10.7164/antibiotics.50.379

[45] Perlatti B., Nichols C. B., Alspaugh J. A., Gloer J. B. & Bills G. F. Sphaerostilbellins, New Antimicrobial Aminolipopeptide Peptaibiotics from Sphaerostilbella toxica. Biomolecules 10, 1371 (2020). 32993102 10.3390/biom10101371 PMC7600149

[46] Brückner H., Fox S. & Degenkolb T. Sequences of Acretocins, Peptaibiotics Containing the Rare 1-Aminocyclopropanecarboxylic Acid, from Acremonium crotocinigenum CBS 217.70. Chem. Biodivers. 16, (2019). 10.1002/cbdv.201900276 31336036

[47] Fredenhagen A., Molleyres L.-P., Böhlendorf B. & Laue G. Structure Determination of Neoefrapeptins A to N: Peptides with Insecticidal Activity Produced by the Fungus Geotrichum candidum. J. Antibiot. (Tokyo) 59, 267–280 (2006). 16883776 10.1038/ja.2006.38

[48] Fujii K., Ikai Y., Oka H., Suzuki M. & Harada K. A Nonempirical Method Using LC/MS for Determination of the Absolute Configuration of Constituent Amino Acids in a Peptide: Combination of Marfey’s Method with Mass Spectrometry and Its Practical Application. Anal. Chem. 69, 5146–5151 (1997).

[49] Vicente-Garcia C. & Colomer I. Lipopeptides as tools in catalysis, supramolecular, materials and medicinal chemistry. Nat. Rev. Chem. 7, 710–731 (2023). 37726383 10.1038/s41570-023-00532-8

[50] Lazzaro B. P., Zasloff M. & Rolff J. Antimicrobial peptides: Application informed by evolution. Science 368, eaau5480 (2020). 32355003 10.1126/science.aau5480 PMC8097767

[51] Wright G. D. Antibiotic Adjuvants: Rescuing Antibiotics from Resistance. Trends Microbiol. 24, 862–871 (2016). 27430191 10.1016/j.tim.2016.06.009

[52] Kim G. H., Rosiana S., Kirienko N. V. & Shapiro R. S. A Simple Nematode Infection Model for Studying Candida albicans Pathogenesis. Curr. Protoc. Microbiol. 59, e114 (2020). 32975912 10.1002/cpmc.114

[53] Revie N. M. Targeting fungal membrane homeostasis with imidazopyrazoindoles impairs azole resistance and biofilm formation. Nat. Commun. 13, 3634 (2022). 35752611 10.1038/s41467-022-31308-1 PMC9233667

[54] Cruz M. R., Graham C. E., Gagliano B. C., Lorenz M. C. & Garsin D. A. Enterococcus faecalis Inhibits Hyphal Morphogenesis and Virulence of Candida albicans. Infect. Immun. 81, 189–200 (2013). 23115035 10.1128/IAI.00914-12 PMC3536143

[55] Maget-Dana R. & Peypoux F. Iturins, a special class of pore-forming lipopeptides: biological and physicochemical properties. Toxicology 87, 151–174 (1994). 8160184 10.1016/0300-483x(94)90159-7

[56] Taylor S. D. & Palmer M. The action mechanism of daptomycin. Bioorg. Med. Chem. 24, 6253–6268 (2016). 27288182 10.1016/j.bmc.2016.05.052

[57] Morikawa M., Hirata Y. & Imanaka T. A study on the structure–function relationship of lipopeptide biosurfactants. Biochim. Biophys. Acta BBA - Mol. Cell Biol. Lipids 1488, 211–218 (2000). 10.1016/s1388-1981(00)00124-4 11082531

[58] Toniolo C. Lipopeptaibols, a novel family of membrane active, antimicrobial peptides. Cell. Mol. Life Sci. CMLS 58, 1179–1188 (2001). 11577977 10.1007/PL00000932 PMC11337402

[59] Karle I. L. Controls exerted by the Aib residue: Helix formation and helix reversal. Biopolymers 60, 351–365 (2001). 12115146 10.1002/1097-0282(2001)60:5<351::AID-BIP10174>3.0.CO;2-U

[60] Ito T., Matsunaga N., Kurashima M., Demizu Y. & Misawa T. Enhancing Chemical Stability through Structural Modification of Antimicrobial Peptides with Non-Proteinogenic Amino Acids. Antibiotics 12, 1326 (2023). 37627746 10.3390/antibiotics12081326 PMC10451648

[61] Biondi B. Conformational properties, membrane interaction, and antibacterial activity of the peptaibiotic chalciporin A: Multitechnique spectroscopic and biophysical investigations on the natural compound and labeled analogs. Pept. Sci. 110, e23083 (2018). 10.1002/bip.23083 29127716

[62] Anderson J. B. Evolution of antifungal-drug resistance: mechanisms and pathogen fitness. Nat. Rev. Microbiol. 3, 547–556 (2005). 15953931 10.1038/nrmicro1179

[63] Xu D. Genome-Wide Fitness Test and Mechanism-of-Action Studies of Inhibitory Compounds in Candida albicans. PLOS Pathog. 3, e92 (2007). 17604452 10.1371/journal.ppat.0030092 PMC1904411

[64] Richter M. F. Predictive compound accumulation rules yield a broad-spectrum antibiotic. Nature 545, 299–304 (2017). 28489819 10.1038/nature22308 PMC5737020

[65] Chen S. C.-A., Slavin M. A. & Sorrell T. C. Echinocandin Antifungal Drugs in Fungal Infections: A Comparison. Drugs 71, 11–41 (2011). 21175238 10.2165/11585270-000000000-00000

[66] Gow N. A. R. & Lenardon M. D. Architecture of the dynamic fungal cell wall. Nat. Rev. Microbiol. 21, 248–259 (2023). 36266346 10.1038/s41579-022-00796-9

[67] Wagener J. & Loiko V. Recent Insights into the Paradoxical Effect of Echinocandins. J. Fungi 4, 5 (2018). 10.3390/jof4010005 PMC5872308 29371498

[68] Walker L. A. Stimulation of Chitin Synthesis Rescues Candida albicans from Echinocandins. PLoS Pathog. 4, e1000040 (2008). 18389063 10.1371/journal.ppat.1000040 PMC2271054

[69] Lee K. K. Yeast species-specific, differential inhibition of β−1,3-glucan synthesis by poacic acid and caspofungin. Cell Surf. 3, 12–25 (2018). 30370375 10.1016/j.tcsw.2018.09.001 PMC6195761

[70] Sudbery P. E. Growth of Candida albicans hyphae. Nat. Rev. Microbiol. 9, 737–748 (2011). 21844880 10.1038/nrmicro2636

[71] Okada H., Ohnuki S., Roncero C., Konopka J. B. & Ohya Y. Distinct roles of cell wall biogenesis in yeast morphogenesis as revealed by multivariate analysis of high-dimensional morphometric data. Mol. Biol. Cell 25, 222–233 (2014). 24258022 10.1091/mbc.E13-07-0396 PMC3890343

[72] El-Kirat-Chatel S. Nanoscale analysis of caspofungin-induced cell surface remodelling in Candida albicans. Nanoscale 5, 1105–1115 (2013). 23262781 10.1039/c2nr33215a PMC3564254

[73] Piotrowski J. S. Plant-derived antifungal agent poacic acid targets β−1,3-glucan. Proc. Natl. Acad. Sci. 112, E1490–E1497 (2015). 25775513 10.1073/pnas.1410400112 PMC4378397

[74] Okada H. & Ohya Y. Fluorescent Labeling of Yeast Cell Wall Components. Cold Spring Harb. Protoc. 2016, (2016). 10.1101/pdb.prot085241 27480714

[75] Aketagawa J., Tanaka S., Tamura H., Shibata Y. & Saitô H. Activation of Limulus Coagulation Factor G by Several (l→3)-β-D-Glucans: Comparison of the Potency of Glucans with Identical Degree of Polymerization but Different Conformations. J. Biochem. (Tokyo) 113, 683–686 (1993). 8370664 10.1093/oxfordjournals.jbchem.a124103

[76] Bizerra F. C. Changes in Cell Wall Synthesis and Ultrastructure during Paradoxical Growth Effect of Caspofungin on Four Different Candida Species. Antimicrob. Agents Chemother. 55, 302–310 (2011). 21060107 10.1128/AAC.00633-10 PMC3019691

[77] Garcia-Rubio R., de Oliveira H. C., Rivera J. & Trevijano-Contador N. The Fungal Cell Wall: Candida, Cryptococcus, and Aspergillus Species. Front. Microbiol. 10, 2993 (2020). 31993032 10.3389/fmicb.2019.02993 PMC6962315

[78] Horton M. V. Candida auris Cell Wall Mannosylation Contributes to Neutrophil Evasion through Pathways Divergent from Candida albicans and Candida glabrata. mSphere 6, 10.1128/msphere.00406-21 (2021). PMC8265655 34160238

[79] Zhao P. Fungi-derived lipopeptide antibiotics developed since 2000. Peptides 113, 52–65 (2019). 30738838 10.1016/j.peptides.2019.02.002

[80] Hoste A. C. R. The structure of lipopeptides impacts their antiviral activity and mode of action against SARS-CoV-2 in vitro. Appl. Environ. Microbiol. 0, e01036–24 (2024). 10.1128/aem.01036-24 PMC11577759 39445780

[81] Grigoriev P. A. Differences in membrane pore formation by peptaibols. J. Pept. Sci. 9, 763–768 (2003). 14658795 10.1002/psc.502

[82] Oh S.-U., Yun B.-S., Lee S.-J., Kim J.-H. & Yoo I.-D. Atroviridins A-C and Neoatroviridins A-D, Novel Peptaibol Antibiotics Produced by Trichoderma atroviride F80317 I. Taxonomy, Fermentation, Isolation and Biological Activities. J. Antibiot. (Tokyo) 55, 557–564 (2002). 12195961 10.7164/antibiotics.55.557

[83] Mootz H. D., Schwarzer D. & Marahiel M. A. Ways of assembling complex natural products on modular nonribosomal peptide synthetases. Chembiochem Eur. J. Chem. Biol. 3, 490–504 (2002). 10.1002/1439-7633(20020603)3:6<490::AID-CBIC490>3.0.CO;2-N 12325005

[84] Blin K. antiSMASH 7.0: new and improved predictions for detection, regulation, chemical structures and visualisation. Nucleic Acids Res. 51, W46–W50 (2023). 37140036 10.1093/nar/gkad344 PMC10320115

[85] Chiang Y.-M. Molecular genetic mining of the Aspergillus secondary metabolome: Discovery of the emericellamide biosynthetic pathway. Chem. Biol. 15, 527–532 (2008). 18559263 10.1016/j.chembiol.2008.05.010 PMC2494592

[86] Bérdy J. Bioactive Microbial Metabolites. J. Antibiot. (Tokyo) 58, 1–26 (2005). 15813176 10.1038/ja.2005.1

[87] Hubrich F. Ribosomally derived lipopeptides containing distinct fatty acyl moieties. Proc. Natl. Acad. Sci. 119, e2113120119 (2022). 35027450 10.1073/pnas.2113120119 PMC8784127

[88] Kraas F. I., Helmetag V., Wittmann M., Strieker M. & Marahiel M. A. Functional Dissection of Surfactin Synthetase Initiation Module Reveals Insights into the Mechanism of Lipoinitiation. Chem. Biol. 17, 872–880 (2010). 20797616 10.1016/j.chembiol.2010.06.015

[89] Ongena M. & Jacques P. Bacillus lipopeptides: versatile weapons for plant disease biocontrol. Trends Microbiol. 16, 115–125 (2008). 18289856 10.1016/j.tim.2007.12.009

[90] Ramachander Turaga V. N. Peptaibols: Antimicrobial Peptides from Fungi. in Bioactive Natural products in Drug Discovery (eds. Singh J., Meshram V. & Gupta M.) 713–730 (Springer, Singapore, 2020). doi:10.1007/978-981-15-1394-7_26.

[91] Gilchrist C. L. M. & Chooi Y.-H. clinker & clustermap.js: automatic generation of gene cluster comparison figures. Bioinforma. Oxf. Engl. 37, 2473–2475 (2021). 10.1093/bioinformatics/btab007 33459763

[92] Hegde V. R. Novel fungal metabolites as cell wall active antifungals: fermentation, isolation, physico-chemical properties, structure and biological activity. J. Antibiot. (Tokyo) 54, 74–83 (2001). 11269717 10.7164/antibiotics.54.74

[93] Mangat C. S., Bharat A., Gehrke S. S. & Brown E. D. Rank Ordering Plate Data Facilitates Data Visualization and Normalization in High-Throughput Screening. SLAS Discov. 19, 1314–1320 (2014). 10.1177/1087057114534298 PMC4318693 24828052

[94] Wang M. Sharing and community curation of mass spectrometry data with Global Natural Products Social Molecular Networking. Nat. Biotechnol. 34, 828–837 (2016). 27504778 10.1038/nbt.3597 PMC5321674

[95] Metin B., Findley K. & Heitman J. The mating type locus (MAT) and sexual reproduction of Cryptococcus heveanensis: insights into the evolution of sex and sex-determining chromosomal regions in fungi. PLoS Genet. 6, e1000961 (2010). 20502678 10.1371/journal.pgen.1000961 PMC2873909

[96] Li H. & Durbin R. Fast and accurate short read alignment with Burrows–Wheeler transform. Bioinformatics 25, 1754–1760 (2009). 19451168 10.1093/bioinformatics/btp324 PMC2705234

[97] Magoč T. & Salzberg S. L. FLASH: fast length adjustment of short reads to improve genome assemblies. Bioinformatics 27, 2957–2963 (2011). 21903629 10.1093/bioinformatics/btr507 PMC3198573

[98] Bankevich A. SPAdes: A New Genome Assembly Algorithm and Its Applications to Single-Cell Sequencing. J. Comput. Biol. 19, 455–477 (2012). 22506599 10.1089/cmb.2012.0021 PMC3342519

[99] Lagesen K. RNAmmer: consistent and rapid annotation of ribosomal RNA genes. Nucleic Acids Res. 35, 3100–3108 (2007). 17452365 10.1093/nar/gkm160 PMC1888812

[100] Altschul S. F., Gish W., Miller W., Myers E. W. & Lipman D. J. Basic local alignment search tool. J. Mol. Biol. 215, 403–410 (1990). 2231712 10.1016/S0022-2836(05)80360-2

[101] Manni M., Berkeley M. R., Seppey M., Simão F. A. & Zdobnov E. M. BUSCO Update: Novel and Streamlined Workflows along with Broader and Deeper Phylogenetic Coverage for Scoring of Eukaryotic, Prokaryotic, and Viral Genomes. Mol. Biol. Evol. 38, 4647–4654 (2021). 34320186 10.1093/molbev/msab199 PMC8476166

[102] Deatherage D. E. & Barrick J. E. Identification of Mutations in Laboratory-Evolved Microbes from Next-Generation Sequencing Data Using breseq. in Engineering and Analyzing Multicellular Systems: Methods and Protocols (eds. Sun L. & Shou W.) 165–188 (Springer, New York, NY, 2014). doi:10.1007/978-1-4939-0554-6_12. PMC4239701 24838886

[103] Fredenhagen A., Molleyres L.-P., Böhlendorf B. & Laue G. Structure Determination of Neoefrapeptins A to N: Peptides with Insecticidal Activity Produced by the Fungus Geotrichum candidum. J. Antibiot. (Tokyo) 59, 267–280 (2006). 16883776 10.1038/ja.2006.38

[104] Okoli I. Identification of Antifungal Compounds Active against Candida albicans Using an Improved High-Throughput Caenorhabditis elegans Assay. PLOS ONE 4, e7025 (2009). 19750012 10.1371/journal.pone.0007025 PMC2737148

[105] Revie N. M. Targeting fungal membrane homeostasis with imidazopyrazoindoles impairs azole resistance and biofilm formation. Nat. Commun. 13, 3634 (2022). 35752611 10.1038/s41467-022-31308-1 PMC9233667

[106] Iyer K. R. Identification of triazenyl indoles as inhibitors of fungal fatty acid biosynthesis with broad-spectrum activity. Cell Chem. Biol. 30, 795–810.e8 (2023). 37369212 10.1016/j.chembiol.2023.06.005 PMC11016341

[107] Stirling D. R. CellProfiler 4: improvements in speed, utility and usability. BMC Bioinformatics 22, 433 (2021). 34507520 10.1186/s12859-021-04344-9 PMC8431850

[108] Schindelin J. Fiji: an open-source platform for biological-image analysis. Nat. Methods 9, 676–682 (2012). 22743772 10.1038/nmeth.2019 PMC3855844

[109] Perrine-Walker F. Caspofungin resistance in Candida albicans: genetic factors and synergistic compounds for combination therapies. Braz. J. Microbiol. 53, 1101–1113 (2022). 35352319 10.1007/s42770-022-00739-9 PMC9433586

[110] Wright R. Transmission electron microscopy of yeast. Microsc. Res. Tech. 51, 496–510 (2000). 11169854 10.1002/1097-0029(20001215)51:6<496::AID-JEMT2>3.0.CO;2-9

[111] Guerra C. R., Ishida K., Nucci M. & Rozental S. Terbinafine inhibits Cryptococcus neoformans growth and modulates fungal morphology. Mem. Inst. Oswaldo Cruz 107, 582–590 (2012). 22850947 10.1590/s0074-02762012000500003

[112] Kovács A. L. The application of traditional transmission electron microscopy for autophagy research in Caenorhabditis elegans. Biophys. Rep. 1, 99–105 (2015). 26942224 10.1007/s41048-015-0014-z PMC4762130

