# Supplementary material for: A microbial natural product fractionation library screen with HRMS/MS dereplication identifies new lipopeptaibiotics against Candida auris
Source: Res Sq. 2025 Jan 17:rs.3.rs-5802877. Preprint. [Version 1] doi: 10.21203/rs.3.rs-5802877/v1 (PMC11774467; doi:10.21203/rs.3.rs-5802877/v1)
Supplement: Supplement 1 — Table 1 is available in the Supplementary Files section. [file NIHPPRS5802877v1-supplement-1.pdf]

b,

Supplementary Files

This is a list of supplementary files associated with this preprint. Click to download.

- Table1.pdf
- ConiotinASupplementaryinformation.pdf
- ConiotinAExtendedData.pdf
